# Supplementary material for: Modeling the demography of species providing extended parental care: A capture–recapture multievent model with a case study on polar bears (Ursus maritimus)
Source: Ecol Evol. 2021 Mar 10;11(7):3380–92. doi: 10.1002/ece3.7296 (PMC8019049; doi:10.1002/ece3.7296)
Supplement: Supplementary file 1 — Appendix S1 [file ECE3-11-3380-s001.pdf]

Appendix 1: Guidance and code to simulate multi-event data based on family units CR histories, with variable litter size and variable timing at offspring independence, and fit the model to the simulated data

Sarah Cubaynes

6/22/2020

All files associated with Appendix 1 are available on GitHub here.

Simulate multi-event data based on family units CR histories

We assume a post-breeding census, with captures occuring in spring (after den exit, cubs are about 4 months old). The year goes from 1st of April year t to 1st of April t+1.

Load useful package :

```
library(jtools)
```

First, set the general parameters used to simulate the data:

```
n.occasions <- 15 # duration of the study
R <- 50 # number of newly family units captured per occasion
nrepet = 100 # number of simulated data set (repetitions)
```

Second, define scenario: S1 (low detection, apha constant=0.5), S2 (low detection alpha varies with date of capture), S3 (high detection, apha constant=0.5) or S4 (low detection alpha varies with date of capture):

```
S <- 2 # set to 1 for S1, 2 for S2, 3 for S3 or 4 for S4

capture <- c(0.25,0.25,0.5,0.5) # capture probability
p <- capture[S]
```

Load the model to infer departure status of two-year old bears at the time of capture (still with family unit or already departed from family unit) . For scenarios S1 and S3, departure probability is constant throughout the field season. For scenarios S2 and S4, we used the regression coefficients estimated from the polar bear data, see Appendix 2.

```
filename <- c("glmdeparture_alphaconstant05.Rdata","glm_departure_date.Rdata",
              "glmdeparture_alphaconstant05.Rdata","glm_departure_date.Rdata")
fileS <- filename[S]
load(file=paste(fileS))

# plot departure rate with date of capture
doypred<-80:130 # day of the year, match the timing of the polar bear field season (spring)
effect_plot(modeld, pred = doy, pred.values=doypred,interval = TRUE,
            plot.points = FALSE,y.label = "departure probability")
```

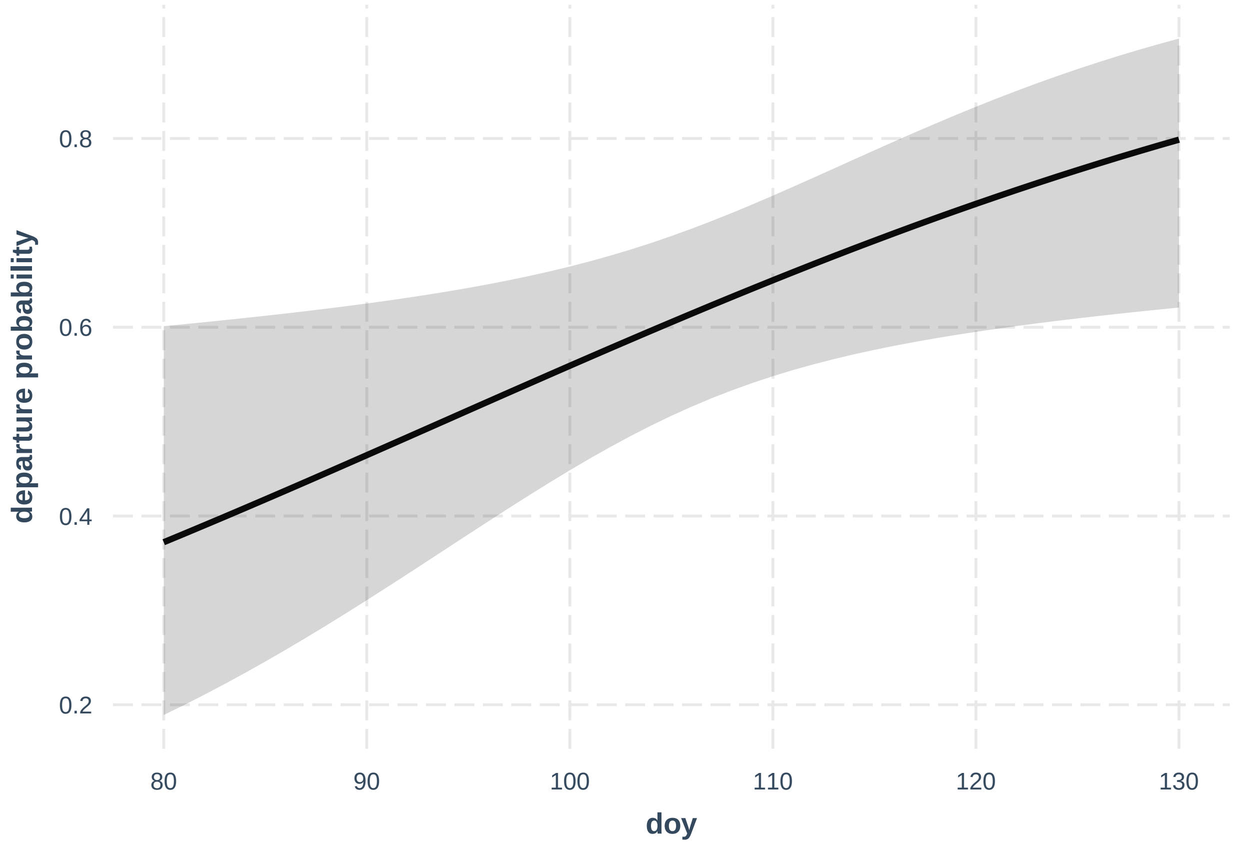

Figure S1. Departure probability of two-year old bears as a fuction of date of capture within the field season in day of the year (doy).

Dates of capture used in the simulations below are randomly sampled from the distribution of capture dates (in day of the year) from the polar bear data:

```
load(file="dateofcapturePB.Rdata") # load data on winter date of capture
hist(wcapt,main="",xlab="Date of capture (doy)") # plot dates of capture
```

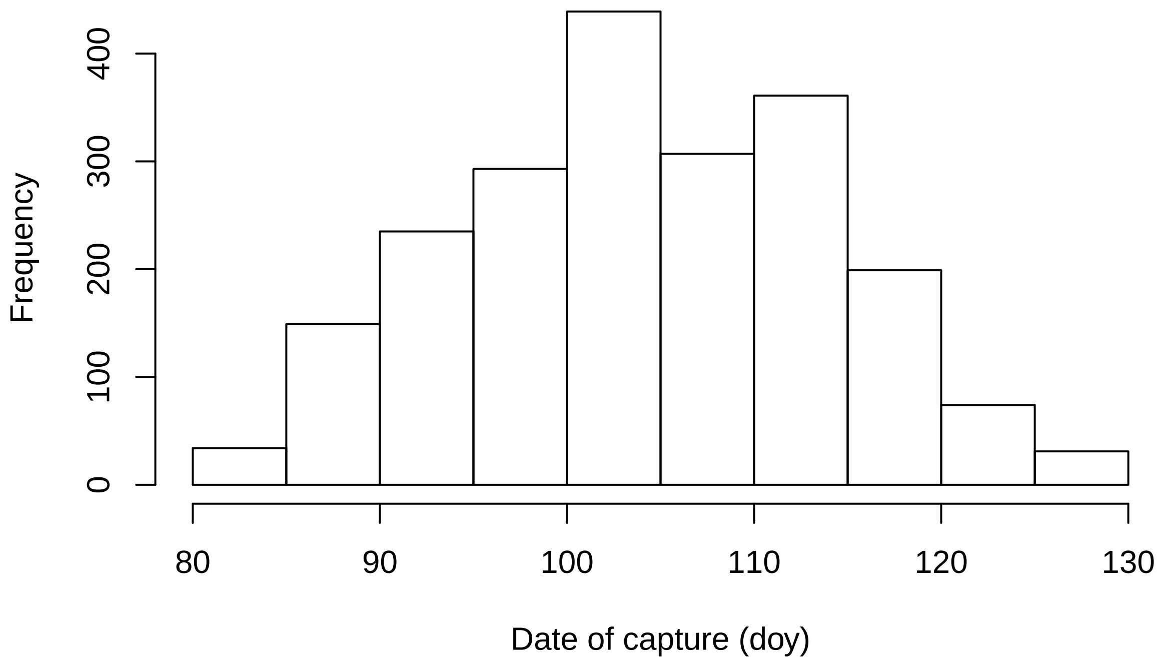

Figure S2. Histogram of te dates of capture of polar bears in Svalbard in day of the year (doy).

Define the demographic parameters used to simulate the data. Here we simulate a virtual long-lived species mimicking the polar bear:

```
phi <- 0.9 # survival of independent juvenile, subadult and adult (phi6+) females.
s <- c(0.6,0.55,0.8,0.75) # dependent offspring survival (conditional upon mother
#survival) by increasing age and litter size (in this order: s1,s2,s3,s4)
# we can calculate litter survival rates from individual offspring survival :
    #Litter survival (lxy, x offspring age, y litter size):
l01 = s[1]
l02 = 1 - (1-s[2]^2-2*s[2]*(1-s[2])) #0.7975for twin litter of cubs
l11= s[3]
l12= 1 - (1-s[4]^2-2*s[4]*(1-s[4]))#0.94 for twin litter of yearlings
    # Probability that :
    #- only one a0 offspring in a litter of 2 survives= 2*s[2]*(1-s[2]),
    #- both survive :s[2]^2
    # Probability that
    #-only one a1 offspring in a litter of 2 survives= 2*s[2]*(1-s[2]),
    #- both offspring survive:s[2]^2
  # Which gives us the transition probabilities for litter size of 1 and 2 offspring,
#that one, both, or any offspring survive:
psi <- c(s[1],2*s[2]*(1-s[2]),s[2]^2,
         (1- s[2]^2 -2*s[2]*(1-s[2])), s[3],2*s[4]*(1-s[4]),s[4]^2,
         1- 2*s[4]*(1-s[4]) - s[4]^2)
# 0.6, 0.495,0.3025,  0.2025, 0.8, 0.375, 0.5625, 0.0625
beta <- c(0.5,0.7,0.9,0.8) # breeding probabilities
#(in the following order beta1,beta2,beta3,beta4)
gamma <- c(0.4,0.5,0.6,0.7) # probability of litter size of 2
#(in the following order gamma1,gamma2,gamma3,gamma4)
```

Define the states and the events used in the model:

```
## Define the states (12 states = 11 alive+ 1dead) describe family unit composition
# as decribed in the methods section
```

```
    # J2  independent 2yo juvenile female
    # J3 independent 3yo juvenile female
    #SA4 independent 4yo subadult female
    #SA5 independent 5yo subadult female
    #A01 adult female caring for 1 dependent offspring of the year (age <1)
    #A02 adult female caring for 2 dependent offspring of the year (age <1)
    #A11 adult female caring for 1 dependent yearling (age 1)
    #A12 adult female caring for 2 dependent yearlings (age 1)
    #AS1 adult female successful breeder raising 1 offspring reaching independence
    #AS2  adult female successful breeder raising 2 offspring reaching independence
    #A adult female without dependent offspring
    # D dead state
n.states <- 11
all.states <- n.states + 1 # with dead state

## Define the events
# as defined in the methods section
    # non-observation code "0"
    # capture of a 2y female code "1"
    # capture of a 3y female code "2"
    # capture of a 4y female code "3"
    # capture of a 5y female code "4"
    # capture of an adult female with 1 offspring of the year (age <1) code "5"
    # capture of an adult female with 2 offspring of the year (age <1) code "6"
    # capture of an adult female with 1 yearling code "7"
    # capture of an adult female with 2 yearlings code "8"
    # capture of an adult female with 1 two-year old offspring code "9"
    # capture of an adult female with 2 two-year old offspring code "10"
    # capture of an adult female alone code "11"
n.events <- 11
all.events <- n.events + 1 #with not seen
```

Define the initial state vector, gathering the proportion of individual in each state at first capture:

```
S0 <- c(rep(1/n.states,n.states),0)#initial state vector S0
```

Here we considered equal proportions in the eleven alive states.

Now construct the transition matrices PHI, PS11, PSI2, PSI3 involved in the state process:

```
### State transitions
# define PHI matrix gathering survival of independent juveniles, subadults and adults
temp1 <- diag(c(phi,phi,phi,phi,rep(phi,7)),nrow=all.states,ncol=n.states)
PHI <- cbind(temp1,c(1-phi,1-phi,1-phi,1-phi,rep(1-phi,7),1))

# Define PSI matrices gathering state-to-state transition probabilities, it includes:

# PSI1: litter survival, individual dependent offspring survival and growth to next age:
PSI1 <- matrix(0,nrow=all.states,ncol=all.states + 1)
for(i in 1:3){PSI1[i,i] <- 1} # juvenile and subadults grow to next age
PSI1[4,12] <- 1 # 5a grow to sexually mature female without dependent offspring
PSI1[5,4] <- psi[1] ; PSI1[5,8] <- (1-psi[1]) #a0 offspring survives or dies
PSI1[6,4] <- psi[2] ; PSI1[6,5] <- psi[3] ; PSI1[6,8] <- psi[4]#1,both
#or any a0 offspring in a litter of 2 survive
PSI1[7,6] <- psi[5] ; PSI1[7,9] <- (1-psi[5])#a1 offspring in a litter
#of 1 survives or die
```

```
PSI1[8,6] <- psi[6] ; PSI1[8,7] <- psi[7] ;PSI1[8,9] <- psi[8]#1,both or any
#a1 offspring in a litter of 2 survive or die
for(i in 9:12){PSI1[i,i+1] <- 1} # for offspring reaching independence and females
#without dependent offspring

# PSI2: breeding probabilities:
PSI2 <- matrix(0,nrow=all.states+1,ncol=all.states+4)
for(i in 1:7){PSI2[i,i] <- 1} # here we assume that juveniles, subaduts and adults
#caring for dependent offspring are not available to breed
PSI2[8,8] <- beta[1];PSI2[8,9] <- 1-beta[1]
PSI2[9,10] <- beta[2];PSI2[9,11] <- 1-beta[2]
PSI2[10,12] <- beta[3];PSI2[10,13] <- 1-beta[3]
PSI2[11,12] <- beta[3];PSI2[11,13] <- 1-beta[3]
PSI2[12,14] <- beta[4];PSI2[12,15] <- 1-beta[4]
PSI2[13,16] <- 1 # dead state

# PSI3:litter size probabilities
#(Pr(singleton litter) = gamma, Pr(twin litter)= 1-gamma)
PSI3 <- matrix(0,nrow=all.states+4,ncol=all.states)
for(i in 1:3){PSI3[i,i+1] <- 1}
for(i in 4:7){PSI3[i,i+3] <- 1}
PSI3[8,5] <- gamma[1];PSI3[8,6] <- 1-gamma[1]
PSI3[9,11] <-1
PSI3[10,5] <- gamma[2];PSI3[10,6] <- 1-gamma[2]
PSI3[11,11] <-1
PSI3[12,5] <- gamma[3];PSI3[12,6] <- 1-gamma[3]
PSI3[13,11] <-1
PSI3[14,5] <- gamma[4];PSI3[14,6] <- 1-gamma[4]
PSI3[15,11] <-1
PSI3[16,12] <-1
```

We can now define the part of the observation process (matrix $E2$ modeling capture probability, matrix $E1$ will be defined later) which does not depend on capture date:

```
# Define observation process
# step 1 : matrix E1
# it involves departure probability which is function of day of capture,
#it will be define below within the loop

# step 2 : matrix E2, involves capture probability
E2 <- matrix(0,nrow=all.states, ncol=all.events)
for(i in 1:all.states){E2[i,i] <- p} #detection probability
E2[,all.events] <- c(rep(1-p,4), rep(1-p,7),1)
```

Below, we generate the data using multinomial trials for each sampling occasion, for each family unit. This script is based on the script from Chapter 7 in Kery and Schaub (2011) to simulate multistate CMR data. To deal with variable timing at offspring independence, the probabilities within matrix $E1$ used in the observation process depend on date of capture. Matrix $E1$ is therefore defined within the loop for each individual at each sampling occasion depending on its date of capture.

```
## Generate data
for(r in 1:nrepet){ # number of dataset generated

  marked <- matrix(0, ncol = all.states, nrow = n.occasions) # empty object to store
  #marked family units
```

```
  for(t in 1:n.occasions){
    marked[t,] <- rmultinom(1,R,prob=S0) # define states at first capture
  }
  # Below we use transition and event matrices wwtih 4-dimensions:
  # Dimension 1: state of departure
  # Dimension 2: state of arrival
  # Dimension 3: family unit
  # Dimension 4: time

  # 1. State process matrix
  totrel <- sum(marked)*(n.occasions-1)
  PSI.STATE <- array(NA, dim=c(all.states, all.states, totrel, n.occasions-1))

  for (i in 1:totrel){
    for (t in 1:(n.occasions-1)){
      PSI.STATE[,,i,t] <- PHI %*% PSI1 %*% PSI2 %*% PSI3 # transition matrix
      #is the matrix product of PHI PSI1 PSI2 and PSI3 defiend above
    } #t
  } #i

  # 2.Observation process matrix
  E <- array(NA, dim=c(all.states, all.events, totrel, n.occasions-1))

  # day of capture, sampled from polar bears capture dates
  daycapt <- matrix(sample(wcapt,size=(totrel*(n.occasions-1)),
                           replace=TRUE), nrow=totrel, ncol=n.occasions-1)

    for (i in 1:totrel){
    for (t in 1:(n.occasions-1)){

  # predict departure probability function of date of capture
  alpha <- predict(modeld,newdata=list(doy=daycapt[i,t]),type="response")

  # now define event matrix E1 using departure probability alpha
  E1 <- diag(1,nrow=all.states,ncol=all.events)
  E1[9,9    ]<- 1-alpha
  E1[9,10]<-    0
  E1[9,11]<-    alpha
  E1[10,9]<-    2*(1-alpha)*alpha
  E1[10,10]<-   1 - (2*(1-alpha)*alpha) - (alpha)^2
  E1[10,11]<-   (alpha)^2

      E[,,i,t] <- E1 %*% E2 # define E which is the matrix product of E1 and E2
    } #t
  } #i
}#r
```

Make a function to simulate the data using the state and event matrices we just defined:

```
  # Define function to simulate multistate capture-recapture data
  simul.ms <- function(PSI.STATE, E, marked, unobservable = NA){
    # Unobservable: number of state that is unobservable
    n.occasions <- dim(PSI.STATE)[4] + 1
    CH <- CH.TRUE <- dayC <- matrix(NA, ncol = n.occasions, nrow = sum(marked))
```

```
  # Define a vector with the occasion of marking
  mark.occ <- matrix(0, ncol = dim(PSI.STATE)[1], nrow = sum(marked))
  g <- colSums(marked)
  for (s in 1:dim(PSI.STATE)[1]){
    if (g[s]==0) next  # To avoid error message if nothing to replace
    mark.occ[(cumsum(g[1:s])-g[s]+1)[s]:cumsum(g[1:s])[s],s] <-
      rep(1:n.occasions, marked[1:n.occasions,s])
  } #s
  for (i in 1:sum(marked)){
    for (s in 1:dim(PSI.STATE)[1]){
      if (mark.occ[i,s]==0) next
      first <- mark.occ[i,s]
      CH[i,first] <- s
      CH.TRUE[i,first] <- s
    } #s
    for (t in (first+1):n.occasions){
      # Multinomial trials for state transitions
      if (first==n.occasions) next
      state <- which(rmultinom(1, 1, PSI.STATE[CH.TRUE[i,t-1],,i,t-1])==1)
      CH.TRUE[i,t] <- state
      # Multinomial trials for observation process
      event <- which(rmultinom(1, 1, E[CH.TRUE[i,t],,i,t-1])==1)
      CH[i,t] <- event
      dayC[i,t] <- daycapt[i,t-1]
    } #t
  } #i
  # Replace the NA and the highest state number (dead) in the file by 0
  CH[is.na(CH)] <- 0
  CH[CH==dim(PSI.STATE)[1]] <- 0
  CH[CH==unobservable] <- 0
  id <- numeric(0)
  for (i in 1:dim(CH)[1]){
    z <- min(which(CH[i,]!=0))
    ifelse(z==dim(CH)[2], id <- c(id,i), id <- c(id))
  }
  return(list(CH=CH[-id,], CH.TRUE=CH.TRUE[-id,],dayC=dayC[-id,]))
  # CH: capture histories to be used
  # CH.TRUE: capture histories with perfect observation
  # dayC: date of capture
}
```

Generate the data using the function above, and save the family units CR histories, underlying states and dates of captures:

```
# Execute function
sim <- simul.ms(PSI.STATE, E, marked)
CH <- sim$CH  # family units CR histories
init <- sim$CH.TRUE # real state matrix used as initial values for jags model
daycapt <- sim$dayC # info on date of capture
# save files
write.table(CH,paste("simCH",r,"_p",p,"_T",n.occasions,".txt",
                     sep=""),row.names=F, col.names=F, sep =" ")
write.table(init,paste("siminit",r,"_p",p,"_T",n.occasions,".txt",
                       sep=""),row.names=F, col.names=F, sep =" ")
```

```
  write.table(daycapt,paste("simdaycapt",r,"_p",p,"_T",n.occasions,".txt",sep=""),
              row.names=F, col.names=F, sep =" ")
}
```

Fit the model to simulated data

First, load the required package:

```
# Load packages
library(jagsUI)
```

```
## Loading required package: lattice
```

```
##
## Attaching package: 'jagsUI'
```

```
## The following object is masked from 'package:utils':
##
##     View
```

Choose working directory name corresponding to scenario S1, S2, S3 or S4 (to read the corresponding data):

```
rep <- c("S1_p025alphaconstant","S2p025alphadate","S3p05alphaconstant","S4p05alphadate")
repname <- rep[1] ## 1 for S1, 2 for S2, 3 for S3, 4 for S4
```

Define capture probability and model to infer departure probability used to simulate the data in the corresponding scenario: S1 (low detection, apha constant=0.5), S2 (low detection alpha varies with date of capture), S3 (high detection, apha constant=0.5) or S4 (low detection alpha varies with date of capture):

```
S <- 2 # set to 1 for S1, 2 for S2, 3 for S3 or 4 for S4

capture <- c(0.25,0.25,0.5,0.5) # capture probability
p <- capture[S]

filename <- c("glmdeparture_alphaconstant05.Rdata","glm_departure_date.Rdata",
              "glmdeparture_alphaconstant05.Rdata","glm_departure_date.Rdata")
fileS <- filename[S]
load(file=paste(fileS))

n.occasions =15 # number of sampling occasions
nrepet = 100 # number of simulated dataset (repetitions)
```

Sink the jags model :

```
# JAGS MODEL
sink("Multieventmodel_Fit_simul.txt")
cat("

model {
  # Probabilities of events given states and states given states

  # vector of initial states
  S0[1] <- prop[1] / (1 + sum(prop[1:10])) # prob. of being in initial state J2
  S0[2] <- prop[2] / (1 + sum(prop[1:10])) # prob. of being in initial state J3
  S0[3] <- prop[3] / (1 + sum(prop[1:10])) # prob. of being in initial state SA4
  S0[4] <- prop[4] / (1 + sum(prop[1:10])) # prob. of being in initial state SA5
  S0[5] <- prop[5] / (1 + sum(prop[1:10])) # prob. of being in initial state A01
```

```
S0[6] <- prop[6] / (1 + sum(prop[1:10])) # prob. of being in initial state A02
S0[7] <- prop[7] / (1 + sum(prop[1:10])) # prob. of being in initial state A11
S0[8] <- prop[8] / (1 + sum(prop[1:10])) # prob. of being in initial state A12
S0[9] <- prop[9] / (1 + sum(prop[1:10])) # prob. of being in initial state AS1
S0[10] <- prop[10] / (1 + sum(prop[1:10])) # prob. of being in initial state AS2
S0[11] <- 1 / (1 + sum(prop[1:10])) # prob. of being in initial state A-
S0[12] <- 0 # prob. of being in initial state dead

# State process: define probabilities of S(t+1) given S(t)
# define PHI matrix gathering survival of independent juveniles, subadults and adults
PHI   [   1   ,   1   ]<- phi
PHI   [   1   ,   2   ]<- 0
PHI   [   1   ,   3   ]<- 0
PHI   [   1   ,   4   ]<- 0
PHI   [   1   ,   5   ]<- 0
PHI   [   1   ,   6   ]<- 0
PHI   [   1   ,   7   ]<- 0
PHI   [   1   ,   8   ]<- 0
PHI   [   1   ,   9   ]<- 0
PHI   [   1   ,   10  ]<- 0
PHI   [   1   ,   11  ]<- 0
PHI   [   1   ,   12  ]<- 1-phi

PHI   [   2   ,   1   ]<- 0
PHI   [   2   ,   2   ]<- phi
PHI   [   2   ,   3   ]<- 0
PHI   [   2   ,   4   ]<- 0
PHI   [   2   ,   5   ]<- 0
PHI   [   2   ,   6   ]<- 0
PHI   [   2   ,   7   ]<- 0
PHI   [   2   ,   8   ]<- 0
PHI   [   2   ,   9   ]<- 0
PHI   [   2   ,   10  ]<- 0
PHI   [   2   ,   11  ]<- 0
PHI   [   2   ,   12  ]<- 1-phi

PHI   [   3   ,   1   ]<- 0
PHI   [   3   ,   2   ]<- 0
PHI   [   3   ,   3   ]<- phi
PHI   [   3   ,   4   ]<- 0
PHI   [   3   ,   5   ]<- 0
PHI   [   3   ,   6   ]<- 0
PHI   [   3   ,   7   ]<- 0
PHI   [   3   ,   8   ]<- 0
PHI   [   3   ,   9   ]<- 0
PHI   [   3   ,   10  ]<- 0
PHI   [   3   ,   11  ]<- 0
PHI   [   3   ,   12  ]<- 1-phi

PHI   [   4   ,   1   ]<- 0
PHI   [   4   ,   2   ]<- 0
PHI   [   4   ,   3   ]<- 0
PHI   [   4   ,   4   ]<- phi
```

```
  PHI   [   4   ,   5   ]<- 0
  PHI   [   4   ,   6   ]<- 0
  PHI   [   4   ,   7   ]<- 0
  PHI   [   4   ,   8   ]<- 0
  PHI   [   4   ,   9   ]<- 0
  PHI   [   4   ,   10  ]<- 0
  PHI   [   4   ,   11  ]<- 0
  PHI   [   4   ,   12  ]<- 1 - phi

  PHI   [   5   ,   1   ]<- 0
  PHI   [   5   ,   2   ]<- 0
  PHI   [   5   ,   3   ]<- 0
  PHI   [   5   ,   4   ]<- 0
  PHI   [   5   ,   5   ]<- phi
  PHI   [   5   ,   6   ]<- 0
  PHI   [   5   ,   7   ]<- 0
  PHI   [   5   ,   8   ]<- 0
  PHI   [   5   ,   9   ]<- 0
  PHI   [   5   ,   10  ]<- 0
  PHI   [   5   ,   11  ]<- 0
  PHI   [   5   ,   12  ]<- 1 - phi

  PHI   [   6   ,   1   ]<- 0
  PHI   [   6   ,   2   ]<- 0
  PHI   [   6   ,   3   ]<- 0
  PHI   [   6   ,   4   ]<- 0
  PHI   [   6   ,   5   ]<- 0
  PHI   [   6   ,   6   ]<- phi
  PHI   [   6   ,   7   ]<- 0
  PHI   [   6   ,   8   ]<- 0
  PHI   [   6   ,   9   ]<- 0
  PHI   [   6   ,   10  ]<- 0
  PHI   [   6   ,   11  ]<- 0
  PHI   [   6   ,   12  ]<- 1 - phi

  PHI   [   7   ,   1   ]<- 0
  PHI   [   7   ,   2   ]<- 0
  PHI   [   7   ,   3   ]<- 0
  PHI   [   7   ,   4   ]<- 0
  PHI   [   7   ,   5   ]<- 0
  PHI   [   7   ,   6   ]<- 0
  PHI   [   7   ,   7   ]<- phi
  PHI   [   7   ,   8   ]<- 0
  PHI   [   7   ,   9   ]<- 0
  PHI   [   7   ,   10  ]<- 0
  PHI   [   7   ,   11  ]<- 0
  PHI   [   7   ,   12  ]<- 1-phi

  PHI   [   8   ,   1   ]<- 0
  PHI   [   8   ,   2   ]<- 0
  PHI   [   8   ,   3   ]<- 0
  PHI   [   8   ,   4   ]<- 0
  PHI   [   8   ,   5   ]<- 0
```

```
PHI   [   8   ,   6   ]<- 0
PHI   [   8   ,   7   ]<- 0
PHI   [   8   ,   8   ]<- phi
PHI   [   8   ,   9   ]<- 0
PHI   [   8   ,   10  ]<- 0
PHI   [   8   ,   11  ]<- 0
PHI   [   8   ,   12  ]<- 1-phi

PHI   [   9   ,   1   ]<- 0
PHI   [   9   ,   2   ]<- 0
PHI   [   9   ,   3   ]<- 0
PHI   [   9   ,   4   ]<- 0
PHI   [   9   ,   5   ]<- 0
PHI   [   9   ,   6   ]<- 0
PHI   [   9   ,   7   ]<- 0
PHI   [   9   ,   8   ]<- 0
PHI   [   9   ,   9   ]<- phi
PHI   [   9   ,   10  ]<- 0
PHI   [   9   ,   11  ]<- 0
PHI   [   9   ,   12  ]<- 1-phi

PHI   [   10  ,   1   ]<- 0
PHI   [   10  ,   2   ]<- 0
PHI   [   10  ,   3   ]<- 0
PHI   [   10  ,   4   ]<- 0
PHI   [   10  ,   5   ]<- 0
PHI   [   10  ,   6   ]<- 0
PHI   [   10  ,   7   ]<- 0
PHI   [   10  ,   8   ]<- 0
PHI   [   10  ,   9   ]<- 0
PHI   [   10  ,   10  ]<- phi
PHI   [   10  ,   11  ]<- 0
PHI   [   10  ,   12  ]<- 1-phi

PHI   [   11  ,   1   ]<- 0
PHI   [   11  ,   2   ]<- 0
PHI   [   11  ,   3   ]<- 0
PHI   [   11  ,   4   ]<- 0
PHI   [   11  ,   5   ]<- 0
PHI   [   11  ,   6   ]<- 0
PHI   [   11  ,   7   ]<- 0
PHI   [   11  ,   8   ]<- 0
PHI   [   11  ,   9   ]<- 0
PHI   [   11  ,   10  ]<- 0
PHI   [   11  ,   11  ]<- phi
PHI   [   11  ,   12  ]<- 1-phi

PHI   [   12  ,   1   ]<- 0
PHI   [   12  ,   2   ]<- 0
PHI   [   12  ,   3   ]<- 0
PHI   [   12  ,   4   ]<- 0
PHI   [   12  ,   5   ]<- 0
PHI   [   12  ,   6   ]<- 0
```

```
PHI   [   12  ,   7   ]<- 0
PHI   [   12  ,   8   ]<- 0
PHI   [   12  ,   9   ]<- 0
PHI   [   12  ,   10  ]<- 0
PHI   [   12  ,   11  ]<- 0
PHI   [   12  ,   12  ]<- 1

# Define PSI matrices gathering state-to-state transition probabilities, it includes:
# PSI1: offspring survival and growth to next age, proba of sexual maturation:
PSI1  [   1   ,   1   ]<- 1
PSI1  [   1   ,   2   ]<- 0
PSI1  [   1   ,   3   ]<- 0
PSI1  [   1   ,   4   ]<- 0
PSI1  [   1   ,   5   ]<- 0
PSI1  [   1   ,   6   ]<- 0
PSI1  [   1   ,   7   ]<- 0
PSI1  [   1   ,   8   ]<- 0
PSI1  [   1   ,   9   ]<- 0
PSI1  [   1   ,   10  ]<- 0
PSI1  [   1   ,   11  ]<- 0
PSI1  [   1   ,   12  ]<- 0
PSI1  [   1   ,   13  ]<- 0

PSI1  [   2   ,   1   ]<- 0
PSI1  [   2   ,   2   ]<- 1
PSI1  [   2   ,   3   ]<- 0
PSI1  [   2   ,   4   ]<- 0
PSI1  [   2   ,   5   ]<- 0
PSI1  [   2   ,   6   ]<- 0
PSI1  [   2   ,   7   ]<- 0
PSI1  [   2   ,   8   ]<- 0
PSI1  [   2   ,   9   ]<- 0
PSI1  [   2   ,   10  ]<- 0
PSI1  [   2   ,   11  ]<- 0
PSI1  [   2   ,   12  ]<- 0
PSI1  [   2   ,   13  ]<- 0

PSI1  [   3   ,   1   ]<- 0
PSI1  [   3   ,   2   ]<- 0
PSI1  [   3   ,   3   ]<- 1-kappa # first repro at age 6
PSI1  [   3   ,   4   ]<- 0
PSI1  [   3   ,   5   ]<- 0
PSI1  [   3   ,   6   ]<- 0
PSI1  [   3   ,   7   ]<- 0
PSI1  [   3   ,   8   ]<- 0
PSI1  [   3   ,   9   ]<- 0
PSI1  [   3   ,   10  ]<- 0
PSI1  [   3   ,   11  ]<- 0
PSI1  [   3   ,   12  ]<- kappa # first repro at age 5
PSI1  [   3   ,   13  ]<- 0

PSI1  [   4   ,   1   ]<- 0
PSI1  [   4   ,   2   ]<- 0
```

```
PSI1  [   4   ,   3   ]<- 0
PSI1  [   4   ,   4   ]<- 0
PSI1  [   4   ,   5   ]<- 0
PSI1  [   4   ,   6   ]<- 0
PSI1  [   4   ,   7   ]<- 0
PSI1  [   4   ,   8   ]<- 0
PSI1  [   4   ,   9   ]<- 0
PSI1  [   4   ,   10  ]<- 0
PSI1  [   4   ,   11  ]<- 0
PSI1  [   4   ,   12  ]<- 1
PSI1  [   4   ,   13  ]<- 0

PSI1  [   5   ,   1   ]<- 0
PSI1  [   5   ,   2   ]<- 0
PSI1  [   5   ,   3   ]<- 0
PSI1  [   5   ,   4   ]<- s[1] # litter of 1, cub's survival
PSI1  [   5   ,   5   ]<- 0
PSI1  [   5   ,   6   ]<- 0
PSI1  [   5   ,   7   ]<- 0
PSI1  [   5   ,   8   ]<- 1-s[1] #litter of 1, cub's death
PSI1  [   5   ,   9   ]<- 0
PSI1  [   5   ,   10  ]<- 0
PSI1  [   5   ,   11  ]<- 0
PSI1  [   5   ,   12  ]<- 0
PSI1  [   5   ,   13  ]<- 0

PSI1  [   6   ,   1   ]<- 0
PSI1  [   6   ,   2   ]<- 0
PSI1  [   6   ,   3   ]<- 0
PSI1  [   6   ,   4   ]<- 2*s[2]*(1-s[2]) # litter of 2, 1 cub survives
PSI1  [   6   ,   5   ]<- s[2]^2 # litter of 2, both cubs survive
PSI1  [   6   ,   6   ]<- 0
PSI1  [   6   ,   7   ]<- 0
PSI1  [   6   ,   8   ]<- (1- s[2]^2 -2*s[2]*(1-s[2])) #litter of 2, both cubs die
PSI1  [   6   ,   9   ]<- 0
PSI1  [   6   ,   10  ]<- 0
PSI1  [   6   ,   11  ]<- 0
PSI1  [   6   ,   12  ]<- 0
PSI1  [   6   ,   13  ]<- 0

PSI1  [   7   ,   1   ]<- 0
PSI1  [   7   ,   2   ]<- 0
PSI1  [   7   ,   3   ]<- 0
PSI1  [   7   ,   4   ]<- 0
PSI1  [   7   ,   5   ]<- 0
PSI1  [   7   ,   6   ]<- s[3] # litter of 1, yearling's survival
PSI1  [   7   ,   7   ]<- 0
PSI1  [   7   ,   8   ]<- 0
PSI1  [   7   ,   9   ]<- (1-s[3] ) # litter of 1, yearling's death
PSI1  [   7   ,   10  ]<- 0
PSI1  [   7   ,   11  ]<- 0
PSI1  [   7   ,   12  ]<- 0
PSI1  [   7   ,   13  ]<- 0
```

```
PSI1  [   8   ,   1   ]<- 0
PSI1  [   8   ,   2   ]<- 0
PSI1  [   8   ,   3   ]<- 0
PSI1  [   8   ,   4   ]<- 0
PSI1  [   8   ,   5   ]<- 0
PSI1  [   8   ,   6   ]<- 2*s[4]*(1-s[4]) # litter of 2, 1 yearling survives
PSI1  [   8   ,   7   ]<- s[4]^2 # litter of 2, both yearlings survive
PSI1  [   8   ,   8   ]<- 0
PSI1  [   8   ,   9   ]<- (1- s[4]^2 -2*s[4]*(1-s[4])) #litter of 2, both yearlings die
PSI1  [   8   ,   10  ]<- 0
PSI1  [   8   ,   11  ]<- 0
PSI1  [   8   ,   12  ]<- 0
PSI1  [   8   ,   13  ]<- 0

PSI1  [   9   ,   1   ]<- 0
PSI1  [   9   ,   2   ]<- 0
PSI1  [   9   ,   3   ]<- 0
PSI1  [   9   ,   4   ]<- 0
PSI1  [   9   ,   5   ]<- 0
PSI1  [   9   ,   6   ]<- 0
PSI1  [   9   ,   7   ]<- 0
PSI1  [   9   ,   8   ]<- 0
PSI1  [   9   ,   9   ]<- 0
PSI1  [   9   ,   10  ]<- 1
PSI1  [   9   ,   11  ]<- 0
PSI1  [   9   ,   12  ]<- 0
PSI1  [   9   ,   13  ]<- 0

PSI1  [   10  ,   1   ]<- 0
PSI1  [   10  ,   2   ]<- 0
PSI1  [   10  ,   3   ]<- 0
PSI1  [   10  ,   4   ]<- 0
PSI1  [   10  ,   5   ]<- 0
PSI1  [   10  ,   6   ]<- 0
PSI1  [   10  ,   7   ]<- 0
PSI1  [   10  ,   8   ]<- 0
PSI1  [   10  ,   9   ]<- 0
PSI1  [   10  ,   10  ]<- 0
PSI1  [   10  ,   11  ]<- 1
PSI1  [   10  ,   12  ]<- 0
PSI1  [   10  ,   13  ]<- 0

PSI1  [   11  ,   1   ]<- 0
PSI1  [   11  ,   2   ]<- 0
PSI1  [   11  ,   3   ]<- 0
PSI1  [   11  ,   4   ]<- 0
PSI1  [   11  ,   5   ]<- 0
PSI1  [   11  ,   6   ]<- 0
PSI1  [   11  ,   7   ]<- 0
PSI1  [   11  ,   8   ]<- 0
PSI1  [   11  ,   9   ]<- 0
PSI1  [   11  ,   10  ]<- 0
PSI1  [   11  ,   11  ]<- 0
```

```
PSI1  [   11  ,   12  ]<- 1
PSI1  [   11  ,   13  ]<- 0

PSI1  [   12  ,   1   ]<- 0
PSI1  [   12  ,   2   ]<- 0
PSI1  [   12  ,   3   ]<- 0
PSI1  [   12  ,   4   ]<- 0
PSI1  [   12  ,   5   ]<- 0
PSI1  [   12  ,   6   ]<- 0
PSI1  [   12  ,   7   ]<- 0
PSI1  [   12  ,   8   ]<- 0
PSI1  [   12  ,   9   ]<- 0
PSI1  [   12  ,   10  ]<- 0
PSI1  [   12  ,   11  ]<- 0
PSI1  [   12  ,   12  ]<- 0
PSI1  [   12  ,   13  ]<- 1

# PSI2: breeding probabilities:
PSI2  [   1   ,   1   ]<- 1
PSI2  [   1   ,   2   ]<- 0
PSI2  [   1   ,   3   ]<- 0
PSI2  [   1   ,   4   ]<- 0
PSI2  [   1   ,   5   ]<- 0
PSI2  [   1   ,   6   ]<- 0
PSI2  [   1   ,   7   ]<- 0
PSI2  [   1   ,   8   ]<- 0
PSI2  [   1   ,   9   ]<- 0
PSI2  [   1   ,   10  ]<- 0
PSI2  [   1   ,   11  ]<- 0
PSI2  [   1   ,   12  ]<- 0
PSI2  [   1   ,   13  ]<- 0
PSI2  [   1   ,   14  ]<- 0
PSI2  [   1   ,   15  ]<- 0
PSI2  [   1   ,   16  ]<- 0

PSI2  [   2   ,   1   ]<- 0
PSI2  [   2   ,   2   ]<- 1
PSI2  [   2   ,   3   ]<- 0
PSI2  [   2   ,   4   ]<- 0
PSI2  [   2   ,   5   ]<- 0
PSI2  [   2   ,   6   ]<- 0
PSI2  [   2   ,   7   ]<- 0
PSI2  [   2   ,   8   ]<- 0
PSI2  [   2   ,   9   ]<- 0
PSI2  [   2   ,   10  ]<- 0
PSI2  [   2   ,   11  ]<- 0
PSI2  [   2   ,   12  ]<- 0
PSI2  [   2   ,   13  ]<- 0
PSI2  [   2   ,   14  ]<- 0
PSI2  [   2   ,   15  ]<- 0
PSI2  [   2   ,   16  ]<- 0

PSI2  [   3   ,   1   ]<- 0
```

```
PSI2  [   3   ,   2   ]<- 0
PSI2  [   3   ,   3   ]<- 1
PSI2  [   3   ,   4   ]<- 0
PSI2  [   3   ,   5   ]<- 0
PSI2  [   3   ,   6   ]<- 0
PSI2  [   3   ,   7   ]<- 0
PSI2  [   3   ,   8   ]<- 0
PSI2  [   3   ,   9   ]<- 0
PSI2  [   3   ,   10  ]<- 0
PSI2  [   3   ,   11  ]<- 0
PSI2  [   3   ,   12  ]<- 0
PSI2  [   3   ,   13  ]<- 0
PSI2  [   3   ,   14  ]<- 0
PSI2  [   3   ,   15  ]<- 0
PSI2  [   3   ,   16  ]<- 0

PSI2  [   4   ,   1   ]<- 0
PSI2  [   4   ,   2   ]<- 0
PSI2  [   4   ,   3   ]<- 0
PSI2  [   4   ,   4   ]<- 1
PSI2  [   4   ,   5   ]<- 0
PSI2  [   4   ,   6   ]<- 0
PSI2  [   4   ,   7   ]<- 0
PSI2  [   4   ,   8   ]<- 0
PSI2  [   4   ,   9   ]<- 0
PSI2  [   4   ,   10  ]<- 0
PSI2  [   4   ,   11  ]<- 0
PSI2  [   4   ,   12  ]<- 0
PSI2  [   4   ,   13  ]<- 0
PSI2  [   4   ,   14  ]<- 0
PSI2  [   4   ,   15  ]<- 0
PSI2  [   4   ,   16  ]<- 0

PSI2  [   5   ,   1   ]<- 0
PSI2  [   5   ,   2   ]<- 0
PSI2  [   5   ,   3   ]<- 0
PSI2  [   5   ,   4   ]<- 0
PSI2  [   5   ,   5   ]<- 1
PSI2  [   5   ,   6   ]<- 0
PSI2  [   5   ,   7   ]<- 0
PSI2  [   5   ,   8   ]<- 0
PSI2  [   5   ,   9   ]<- 0
PSI2  [   5   ,   10  ]<- 0
PSI2  [   5   ,   11  ]<- 0
PSI2  [   5   ,   12  ]<- 0
PSI2  [   5   ,   13  ]<- 0
PSI2  [   5   ,   14  ]<- 0
PSI2  [   5   ,   15  ]<- 0
PSI2  [   5   ,   16  ]<- 0

PSI2  [   6   ,   1   ]<- 0
PSI2  [   6   ,   2   ]<- 0
PSI2  [   6   ,   3   ]<- 0
```

```
PSI2  [   6   ,   4   ]<- 0
PSI2  [   6   ,   5   ]<- 0
PSI2  [   6   ,   6   ]<- 1
PSI2  [   6   ,   7   ]<- 0
PSI2  [   6   ,   8   ]<- 0
PSI2  [   6   ,   9   ]<- 0
PSI2  [   6   ,   10  ]<- 0
PSI2  [   6   ,   11  ]<- 0
PSI2  [   6   ,   12  ]<- 0
PSI2  [   6   ,   13  ]<- 0
PSI2  [   6   ,   14  ]<- 0
PSI2  [   6   ,   15  ]<- 0
PSI2  [   6   ,   16  ]<- 0

PSI2  [   7   ,   1   ]<- 0
PSI2  [   7   ,   2   ]<- 0
PSI2  [   7   ,   3   ]<- 0
PSI2  [   7   ,   4   ]<- 0
PSI2  [   7   ,   5   ]<- 0
PSI2  [   7   ,   6   ]<- 0
PSI2  [   7   ,   7   ]<- 1
PSI2  [   7   ,   8   ]<- 0
PSI2  [   7   ,   9   ]<- 0
PSI2  [   7   ,   10  ]<- 0
PSI2  [   7   ,   11  ]<- 0
PSI2  [   7   ,   12  ]<- 0
PSI2  [   7   ,   13  ]<- 0
PSI2  [   7   ,   14  ]<- 0
PSI2  [   7   ,   15  ]<- 0
PSI2  [   7   ,   16  ]<- 0

PSI2  [   8   ,   1   ]<- 0
PSI2  [   8   ,   2   ]<- 0
PSI2  [   8   ,   3   ]<- 0
PSI2  [   8   ,   4   ]<- 0
PSI2  [   8   ,   5   ]<- 0
PSI2  [   8   ,   6   ]<- 0
PSI2  [   8   ,   7   ]<- 0
PSI2  [   8   ,   8   ]<- beta[1]
PSI2  [   8   ,   9   ]<- 1-beta[1]
PSI2  [   8   ,   10  ]<- 0
PSI2  [   8   ,   11  ]<- 0
PSI2  [   8   ,   12  ]<- 0
PSI2  [   8   ,   13  ]<- 0
PSI2  [   8   ,   14  ]<- 0
PSI2  [   8   ,   15  ]<- 0
PSI2  [   8   ,   16  ]<- 0

PSI2  [   9   ,   1   ]<- 0
PSI2  [   9   ,   2   ]<- 0
PSI2  [   9   ,   3   ]<- 0
PSI2  [   9   ,   4   ]<- 0
PSI2  [   9   ,   5   ]<- 0
```

```
PSI2  [   9   ,   6   ]<- 0
PSI2  [   9   ,   7   ]<- 0
PSI2  [   9   ,   8   ]<- 0
PSI2  [   9   ,   9   ]<- 0
PSI2  [   9   ,   10  ]<- beta[2]
PSI2  [   9   ,   11  ]<- 1-beta[2]
PSI2  [   9   ,   12  ]<- 0
PSI2  [   9   ,   13  ]<- 0
PSI2  [   9   ,   14  ]<- 0
PSI2  [   9   ,   15  ]<- 0
PSI2  [   9   ,   16  ]<- 0

PSI2  [   10  ,   1   ]<- 0
PSI2  [   10  ,   2   ]<- 0
PSI2  [   10  ,   3   ]<- 0
PSI2  [   10  ,   4   ]<- 0
PSI2  [   10  ,   5   ]<- 0
PSI2  [   10  ,   6   ]<- 0
PSI2  [   10  ,   7   ]<- 0
PSI2  [   10  ,   8   ]<- 0
PSI2  [   10  ,   9   ]<- 0
PSI2  [   10  ,   10  ]<- 0
PSI2  [   10  ,   11  ]<- 0
PSI2  [   10  ,   12  ]<- beta[3]
PSI2  [   10  ,   13  ]<- 1-beta[3]
PSI2  [   10  ,   14  ]<- 0
PSI2  [   10  ,   15  ]<- 0
PSI2  [   10  ,   16  ]<- 0

PSI2  [   11  ,   1   ]<- 0
PSI2  [   11  ,   2   ]<- 0
PSI2  [   11  ,   3   ]<- 0
PSI2  [   11  ,   4   ]<- 0
PSI2  [   11  ,   5   ]<- 0
PSI2  [   11  ,   6   ]<- 0
PSI2  [   11  ,   7   ]<- 0
PSI2  [   11  ,   8   ]<- 0
PSI2  [   11  ,   9   ]<- 0
PSI2  [   11  ,   10  ]<- 0
PSI2  [   11  ,   11  ]<- 0
PSI2  [   11  ,   12  ]<- beta[3]
PSI2  [   11  ,   13  ]<- 1-beta[3]
PSI2  [   11  ,   14  ]<- 0
PSI2  [   11  ,   15  ]<- 0
PSI2  [   11  ,   16  ]<- 0

PSI2  [   12  ,   1   ]<- 0
PSI2  [   12  ,   2   ]<- 0
PSI2  [   12  ,   3   ]<- 0
PSI2  [   12  ,   4   ]<- 0
PSI2  [   12  ,   5   ]<- 0
PSI2  [   12  ,   6   ]<- 0
PSI2  [   12  ,   7   ]<- 0
```

```
PSI2  [   12  ,   8   ]<- 0
PSI2  [   12  ,   9   ]<- 0
PSI2  [   12  ,   10  ]<- 0
PSI2  [   12  ,   11  ]<- 0
PSI2  [   12  ,   12  ]<- 0
PSI2  [   12  ,   13  ]<- 0
PSI2  [   12  ,   14  ]<- beta[4]
PSI2  [   12  ,   15  ]<- 1-beta[4]
PSI2  [   12  ,   16  ]<- 0

PSI2  [   13  ,   1   ]<- 0
PSI2  [   13  ,   2   ]<- 0
PSI2  [   13  ,   3   ]<- 0
PSI2  [   13  ,   4   ]<- 0
PSI2  [   13  ,   5   ]<- 0
PSI2  [   13  ,   6   ]<- 0
PSI2  [   13  ,   7   ]<- 0
PSI2  [   13  ,   8   ]<- 0
PSI2  [   13  ,   9   ]<- 0
PSI2  [   13  ,   10  ]<- 0
PSI2  [   13  ,   11  ]<- 0
PSI2  [   13  ,   12  ]<- 0
PSI2  [   13  ,   13  ]<- 0
PSI2  [   13  ,   14  ]<- 0
PSI2  [   13  ,   15  ]<- 0
PSI2  [   13  ,   16  ]<- 1

# PSI3:litter size probabilities
PSI3  [   1   ,   1   ]<- 0
PSI3  [   1   ,   2   ]<- 1
PSI3  [   1   ,   3   ]<- 0
PSI3  [   1   ,   4   ]<- 0
PSI3  [   1   ,   5   ]<- 0
PSI3  [   1   ,   6   ]<- 0
PSI3  [   1   ,   7   ]<- 0
PSI3  [   1   ,   8   ]<- 0
PSI3  [   1   ,   9   ]<- 0
PSI3  [   1   ,   10  ]<- 0
PSI3  [   1   ,   11  ]<- 0
PSI3  [   1   ,   12  ]<- 0

PSI3  [   2   ,   1   ]<- 0
PSI3  [   2   ,   2   ]<- 0
PSI3  [   2   ,   3   ]<- 1
PSI3  [   2   ,   4   ]<- 0
PSI3  [   2   ,   5   ]<- 0
PSI3  [   2   ,   6   ]<- 0
PSI3  [   2   ,   7   ]<- 0
PSI3  [   2   ,   8   ]<- 0
PSI3  [   2   ,   9   ]<- 0
PSI3  [   2   ,   10  ]<- 0
PSI3  [   2   ,   11  ]<- 0
PSI3  [   2   ,   12  ]<- 0
```

```
PSI3  [   3   ,   1   ]<- 0
PSI3  [   3   ,   2   ]<- 0
PSI3  [   3   ,   3   ]<- 0
PSI3  [   3   ,   4   ]<- 1
PSI3  [   3   ,   5   ]<- 0
PSI3  [   3   ,   6   ]<- 0
PSI3  [   3   ,   7   ]<- 0
PSI3  [   3   ,   8   ]<- 0
PSI3  [   3   ,   9   ]<- 0
PSI3  [   3   ,   10  ]<- 0
PSI3  [   3   ,   11  ]<- 0
PSI3  [   3   ,   12  ]<- 0

PSI3  [   4   ,   1   ]<- 0
PSI3  [   4   ,   2   ]<- 0
PSI3  [   4   ,   3   ]<- 0
PSI3  [   4   ,   4   ]<- 0
PSI3  [   4   ,   5   ]<- 0
PSI3  [   4   ,   6   ]<- 0
PSI3  [   4   ,   7   ]<- 1
PSI3  [   4   ,   8   ]<- 0
PSI3  [   4   ,   9   ]<- 0
PSI3  [   4   ,   10  ]<- 0
PSI3  [   4   ,   11  ]<- 0
PSI3  [   4   ,   12  ]<- 0

PSI3  [   5   ,   1   ]<- 0
PSI3  [   5   ,   2   ]<- 0
PSI3  [   5   ,   3   ]<- 0
PSI3  [   5   ,   4   ]<- 0
PSI3  [   5   ,   5   ]<- 0
PSI3  [   5   ,   6   ]<- 0
PSI3  [   5   ,   7   ]<- 0
PSI3  [   5   ,   8   ]<- 1
PSI3  [   5   ,   9   ]<- 0
PSI3  [   5   ,   10  ]<- 0
PSI3  [   5   ,   11  ]<- 0
PSI3  [   5   ,   12  ]<- 0

PSI3  [   6   ,   1   ]<- 0
PSI3  [   6   ,   2   ]<- 0
PSI3  [   6   ,   3   ]<- 0
PSI3  [   6   ,   4   ]<- 0
PSI3  [   6   ,   5   ]<- 0
PSI3  [   6   ,   6   ]<- 0
PSI3  [   6   ,   7   ]<- 0
PSI3  [   6   ,   8   ]<- 0
PSI3  [   6   ,   9   ]<- 1
PSI3  [   6   ,   10  ]<- 0
PSI3  [   6   ,   11  ]<- 0
PSI3  [   6   ,   12  ]<- 0

PSI3  [   7   ,   1   ]<- 0
```

```
PSI3  [   7   ,   2   ]<- 0
PSI3  [   7   ,   3   ]<- 0
PSI3  [   7   ,   4   ]<- 0
PSI3  [   7   ,   5   ]<- 0
PSI3  [   7   ,   6   ]<- 0
PSI3  [   7   ,   7   ]<- 0
PSI3  [   7   ,   8   ]<- 0
PSI3  [   7   ,   9   ]<- 0
PSI3  [   7   ,   10  ]<- 1
PSI3  [   7   ,   11  ]<- 0
PSI3  [   7   ,   12  ]<- 0

PSI3  [   8   ,   1   ]<- 0
PSI3  [   8   ,   2   ]<- 0
PSI3  [   8   ,   3   ]<- 0
PSI3  [   8   ,   4   ]<- 0
PSI3  [   8   ,   5   ]<- gamma[1]
PSI3  [   8   ,   6   ]<- 1-gamma[1]
PSI3  [   8   ,   7   ]<- 0
PSI3  [   8   ,   8   ]<- 0
PSI3  [   8   ,   9   ]<- 0
PSI3  [   8   ,   10  ]<- 0
PSI3  [   8   ,   11  ]<- 0
PSI3  [   8   ,   12  ]<- 0

PSI3  [   9   ,   1   ]<- 0
PSI3  [   9   ,   2   ]<- 0
PSI3  [   9   ,   3   ]<- 0
PSI3  [   9   ,   4   ]<- 0
PSI3  [   9   ,   5   ]<- 0
PSI3  [   9   ,   6   ]<- 0
PSI3  [   9   ,   7   ]<- 0
PSI3  [   9   ,   8   ]<- 0
PSI3  [   9   ,   9   ]<- 0
PSI3  [   9   ,   10  ]<- 0
PSI3  [   9   ,   11  ]<- 1
PSI3  [   9   ,   12  ]<- 0

PSI3  [   10  ,   1   ]<- 0
PSI3  [   10  ,   2   ]<- 0
PSI3  [   10  ,   3   ]<- 0
PSI3  [   10  ,   4   ]<- 0
PSI3  [   10  ,   5   ]<- gamma[2]
PSI3  [   10  ,   6   ]<-  1-gamma[2]
PSI3  [   10  ,   7   ]<- 0
PSI3  [   10  ,   8   ]<- 0
PSI3  [   10  ,   9   ]<- 0
PSI3  [   10  ,   10  ]<- 0
PSI3  [   10  ,   11  ]<- 0
PSI3  [   10  ,   12  ]<- 0

PSI3  [   11  ,   1   ]<- 0
PSI3  [   11  ,   2   ]<- 0
```

```
PSI3  [   11  ,   3   ]<- 0
PSI3  [   11  ,   4   ]<- 0
PSI3  [   11  ,   5   ]<- 0
PSI3  [   11  ,   6   ]<- 0
PSI3  [   11  ,   7   ]<- 0
PSI3  [   11  ,   8   ]<- 0
PSI3  [   11  ,   9   ]<- 0
PSI3  [   11  ,   10  ]<- 0
PSI3  [   11  ,   11  ]<- 1
PSI3  [   11  ,   12  ]<- 0

PSI3  [   12  ,   1   ]<- 0
PSI3  [   12  ,   2   ]<- 0
PSI3  [   12  ,   3   ]<- 0
PSI3  [   12  ,   4   ]<- 0
PSI3  [   12  ,   5   ]<- gamma[3]
PSI3  [   12  ,   6   ]<- 1-gamma[3]
PSI3  [   12  ,   7   ]<- 0
PSI3  [   12  ,   8   ]<- 0
PSI3  [   12  ,   9   ]<- 0
PSI3  [   12  ,   10  ]<- 0
PSI3  [   12  ,   11  ]<- 0
PSI3  [   12  ,   12  ]<- 0

PSI3  [   13  ,   1   ]<- 0
PSI3  [   13  ,   2   ]<- 0
PSI3  [   13  ,   3   ]<- 0
PSI3  [   13  ,   4   ]<- 0
PSI3  [   13  ,   5   ]<- 0
PSI3  [   13  ,   6   ]<- 0
PSI3  [   13  ,   7   ]<- 0
PSI3  [   13  ,   8   ]<- 0
PSI3  [   13  ,   9   ]<- 0
PSI3  [   13  ,   10  ]<- 0
PSI3  [   13  ,   11  ]<- 1
PSI3  [   13  ,   12  ]<- 0

PSI3  [   14  ,   1   ]<- 0
PSI3  [   14  ,   2   ]<- 0
PSI3  [   14  ,   3   ]<- 0
PSI3  [   14  ,   4   ]<- 0
PSI3  [   14  ,   5   ]<- gamma[4]
PSI3  [   14  ,   6   ]<- 1-gamma[4]
PSI3  [   14  ,   7   ]<- 0
PSI3  [   14  ,   8   ]<- 0
PSI3  [   14  ,   9   ]<- 0
PSI3  [   14  ,   10  ]<- 0
PSI3  [   14  ,   11  ]<- 0
PSI3  [   14  ,   12  ]<- 0

PSI3  [   15  ,   1   ]<- 0
PSI3  [   15  ,   2   ]<- 0
PSI3  [   15  ,   3   ]<- 0
```

```
PSI3  [   15  ,   4   ]<- 0
PSI3  [   15  ,   5   ]<- 0
PSI3  [   15  ,   6   ]<- 0
PSI3  [   15  ,   7   ]<- 0
PSI3  [   15  ,   8   ]<- 0
PSI3  [   15  ,   9   ]<- 0
PSI3  [   15  ,   10  ]<- 0
PSI3  [   15  ,   11  ]<- 1
PSI3  [   15  ,   12  ]<- 0

PSI3  [   16  ,   1   ]<- 0
PSI3  [   16  ,   2   ]<- 0
PSI3  [   16  ,   3   ]<- 0
PSI3  [   16  ,   4   ]<- 0
PSI3  [   16  ,   5   ]<- 0
PSI3  [   16  ,   6   ]<- 0
PSI3  [   16  ,   7   ]<- 0
PSI3  [   16  ,   8   ]<- 0
PSI3  [   16  ,   9   ]<- 0
PSI3  [   16  ,   10  ]<- 0
PSI3  [   16  ,   11  ]<- 0
PSI3  [   16  ,   12  ]<- 1

# Matrix product for state-to-state transitions S
S[1:12,1:12] <- PHI[1:12,1:12] %*% PSI1[1:12,1:13] %*% PSI2[1:13,1:16]  %*% PSI3[1:16,1:12]


## Observation process: Define probabilities of E(t) given S(t).

#for initial capture, conditional on first capture
E0    [   1   ,   1   ]<- 0
E0    [   1   ,   2   ]<- 1
E0    [   1   ,   3   ]<- 0
E0    [   1   ,   4   ]<- 0
E0    [   1   ,   5   ]<- 0
E0    [   1   ,   6   ]<- 0
E0    [   1   ,   7   ]<- 0
E0    [   1   ,   8   ]<- 0
E0    [   1   ,   9   ]<- 0
E0    [   1   ,   10  ]<- 0
E0    [   1   ,   11  ]<- 0
E0    [   1   ,   12  ]<- 0

E0    [   2   ,   1   ]<- 0
E0    [   2   ,   2   ]<- 0
E0    [   2   ,   3   ]<- 1
E0    [   2   ,   4   ]<- 0
E0    [   2   ,   5   ]<- 0
E0    [   2   ,   6   ]<- 0
E0    [   2   ,   7   ]<- 0
E0    [   2   ,   8   ]<- 0
E0    [   2   ,   9   ]<- 0
E0    [   2   ,   10  ]<- 0
```

```
E0    [   2   ,   11  ]<- 0
E0    [   2   ,   12  ]<- 0

E0    [   3   ,   1   ]<- 0
E0    [   3   ,   2   ]<- 0
E0    [   3   ,   3   ]<- 0
E0    [   3   ,   4   ]<- 1
E0    [   3   ,   5   ]<- 0
E0    [   3   ,   6   ]<- 0
E0    [   3   ,   7   ]<- 0
E0    [   3   ,   8   ]<- 0
E0    [   3   ,   9   ]<- 0
E0    [   3   ,   10  ]<- 0
E0    [   3   ,   11  ]<- 0
E0    [   3   ,   12  ]<- 0

E0    [   4   ,   1   ]<- 0
E0    [   4   ,   2   ]<- 0
E0    [   4   ,   3   ]<- 0
E0    [   4   ,   4   ]<- 0
E0    [   4   ,   5   ]<- 1
E0    [   4   ,   6   ]<- 0
E0    [   4   ,   7   ]<- 0
E0    [   4   ,   8   ]<- 0
E0    [   4   ,   9   ]<- 0
E0    [   4   ,   10  ]<- 0
E0    [   4   ,   11  ]<- 0
E0    [   4   ,   12  ]<- 0

E0    [   5   ,   1   ]<- 0
E0    [   5   ,   2   ]<- 0
E0    [   5   ,   3   ]<- 0
E0    [   5   ,   4   ]<- 0
E0    [   5   ,   5   ]<- 0
E0    [   5   ,   6   ]<- 1
E0    [   5   ,   7   ]<- 0
E0    [   5   ,   8   ]<- 0
E0    [   5   ,   9   ]<- 0
E0    [   5   ,   10  ]<- 0
E0    [   5   ,   11  ]<- 0
E0    [   5   ,   12  ]<- 0

E0    [   6   ,   1   ]<- 0
E0    [   6   ,   2   ]<- 0
E0    [   6   ,   3   ]<- 0
E0    [   6   ,   4   ]<- 0
E0    [   6   ,   5   ]<- 0
E0    [   6   ,   6   ]<- 0
E0    [   6   ,   7   ]<- 1
E0    [   6   ,   8   ]<- 0
E0    [   6   ,   9   ]<- 0
E0    [   6   ,   10  ]<- 0
E0    [   6   ,   11  ]<- 0
```

```
E0    [   6   ,   12  ]<- 0

E0    [   7   ,   1   ]<- 0
E0    [   7   ,   2   ]<- 0
E0    [   7   ,   3   ]<- 0
E0    [   7   ,   4   ]<- 0
E0    [   7   ,   5   ]<- 0
E0    [   7   ,   6   ]<- 0
E0    [   7   ,   7   ]<- 0
E0    [   7   ,   8   ]<- 1
E0    [   7   ,   9   ]<- 0
E0    [   7   ,   10  ]<- 0
E0    [   7   ,   11  ]<- 0
E0    [   7   ,   12  ]<- 0

E0    [   8   ,   1   ]<- 0
E0    [   8   ,   2   ]<- 0
E0    [   8   ,   3   ]<- 0
E0    [   8   ,   4   ]<- 0
E0    [   8   ,   5   ]<- 0
E0    [   8   ,   6   ]<- 0
E0    [   8   ,   7   ]<- 0
E0    [   8   ,   8   ]<- 0
E0    [   8   ,   9   ]<- 1
E0    [   8   ,   10  ]<- 0
E0    [   8   ,   11  ]<- 0
E0    [   8   ,   12  ]<- 0

E0    [   9   ,   1   ]<- 0
E0    [   9   ,   2   ]<- 0
E0    [   9   ,   3   ]<- 0
E0    [   9   ,   4   ]<- 0
E0    [   9   ,   5   ]<- 0
E0    [   9   ,   6   ]<- 0
E0    [   9   ,   7   ]<- 0
E0    [   9   ,   8   ]<- 0
E0    [   9   ,   9   ]<- 0
E0    [   9   ,   10  ]<- 1
E0    [   9   ,   11  ]<- 0
E0    [   9   ,   12  ]<- 0

E0    [   10  ,   1   ]<- 0
E0    [   10  ,   2   ]<- 0
E0    [   10  ,   3   ]<- 0
E0    [   10  ,   4   ]<- 0
E0    [   10  ,   5   ]<- 0
E0    [   10  ,   6   ]<- 0
E0    [   10  ,   7   ]<- 0
E0    [   10  ,   8   ]<- 0
E0    [   10  ,   9   ]<- 0
E0    [   10  ,   10  ]<- 0
E0    [   10  ,   11  ]<- 1
E0    [   10  ,   12  ]<- 0
```

```
E0    [   11  ,   1   ]<- 0
E0    [   11  ,   2   ]<- 0
E0    [   11  ,   3   ]<- 0
E0    [   11  ,   4   ]<- 0
E0    [   11  ,   5   ]<- 0
E0    [   11  ,   6   ]<- 0
E0    [   11  ,   7   ]<- 0
E0    [   11  ,   8   ]<- 0
E0    [   11  ,   9   ]<- 0
E0    [   11  ,   10  ]<- 0
E0    [   11  ,   11  ]<- 0
E0    [   11  ,   12  ]<- 1

E0    [   12  ,   1   ]<- 1
E0    [   12  ,   2   ]<- 0
E0    [   12  ,   3   ]<- 0
E0    [   12  ,   4   ]<- 0
E0    [   12  ,   5   ]<- 0
E0    [   12  ,   6   ]<- 0
E0    [   12  ,   7   ]<- 0
E0    [   12  ,   8   ]<- 0
E0    [   12  ,   9   ]<- 0
E0    [   12  ,   10  ]<- 0
E0    [   12  ,   11  ]<- 0
E0    [   12  ,   12  ]<- 0

# departure probability of a2 offspring
for(i in 1:N){
  for(t in 1:(Years-1)){
    E1    [   1   ,   1   ,i,t]<- 1
    E1    [   1   ,   2   ,i,t]<- 0
    E1    [   1   ,   3   ,i,t]<- 0
    E1    [   1   ,   4   ,i,t]<- 0
    E1    [   1   ,   5   ,i,t]<- 0
    E1    [   1   ,   6   ,i,t]<- 0
    E1    [   1   ,   7   ,i,t]<- 0
    E1    [   1   ,   8   ,i,t]<- 0
    E1    [   1   ,   9   ,i,t]<- 0
    E1    [   1   ,   10,i,t]<-   0
    E1    [   1   ,   11,i,t]<-   0
    E1    [   1   ,   12,i,t]<-   0

    E1    [   2   ,   1   ,i,t]<- 0
    E1    [   2   ,   2   ,i,t]<- 1
    E1    [   2   ,   3   ,i,t]<- 0
    E1    [   2   ,   4   ,i,t]<- 0
    E1    [   2   ,   5   ,i,t]<- 0
    E1    [   2   ,   6   ,i,t]<- 0
    E1    [   2   ,   7   ,i,t]<- 0
    E1    [   2   ,   8   ,i,t]<- 0
    E1    [   2   ,   9   ,i,t]<- 0
    E1    [   2   ,   10,i,t]<-   0
    E1    [   2   ,   11,i,t]<-   0
```

```
    E1    [   2   ,   12,i,t]<-   0

    E1    [   3   ,   1   ,i,t]<- 0
    E1    [   3   ,   2   ,i,t]<- 0
    E1    [   3   ,   3   ,i,t]<- 1
    E1    [   3   ,   4   ,i,t]<- 0
    E1    [   3   ,   5   ,i,t]<- 0
    E1    [   3   ,   6   ,i,t]<- 0
    E1    [   3   ,   7   ,i,t]<- 0
    E1    [   3   ,   8   ,i,t]<- 0
    E1    [   3   ,   9   ,i,t]<- 0
    E1    [   3   ,   10,i,t]<-   0
    E1    [   3   ,   11,i,t]<-   0
    E1    [   3   ,   12,i,t]<-   0

    E1    [   4   ,   1   ,i,t]<- 0
    E1    [   4   ,   2   ,i,t]<- 0
    E1    [   4   ,   3   ,i,t]<- 0
    E1    [   4   ,   4   ,i,t]<- 1
    E1    [   4   ,   5   ,i,t]<- 0
    E1    [   4   ,   6   ,i,t]<- 0
    E1    [   4   ,   7   ,i,t]<- 0
    E1    [   4   ,   8   ,i,t]<- 0
    E1    [   4   ,   9   ,i,t]<- 0
    E1    [   4   ,   10,i,t]<-   0
    E1    [   4   ,   11,i,t]<-   0
    E1    [   4   ,   12,i,t]<-   0

    E1    [   5   ,   1,i,t]<-    0
    E1    [   5   ,   2,i,t]<-    0
    E1    [   5   ,   3,i,t]<-    0
    E1    [   5   ,   4,i,t]<-    0
    E1    [   5   ,   5,i,t]<-    1
    E1    [   5   ,   6,i,t]<-    0
    E1    [   5   ,   7,i,t]<-    0
    E1    [   5   ,   8,i,t]<-    0
    E1    [   5   ,   9,i,t]<-    0
    E1    [   5   ,   10,i,t]<-   0
    E1    [   5   ,   11,i,t]<-   0
    E1    [   5   ,   12,i,t]<-   0

    E1    [   6   ,   1   ,i,t]<- 0
    E1    [   6   ,   2   ,i,t]<- 0
    E1    [   6   ,   3   ,i,t]<- 0
    E1    [   6   ,   4   ,i,t]<- 0
    E1    [   6   ,   5   ,i,t]<- 0
    E1    [   6   ,   6   ,i,t]<- 1
    E1    [   6   ,   7   ,i,t]<- 0
    E1    [   6   ,   8   ,i,t]<- 0
    E1    [   6   ,   9   ,i,t]<- 0
    E1    [   6   ,   10,i,t]<-   0
    E1    [   6   ,   11,i,t]<-   0
    E1    [   6   ,   12,i,t]<-   0
```

```
E1    [   7   ,   1   ,i,t]<- 0
E1    [   7   ,   2   ,i,t]<- 0
E1    [   7   ,   3   ,i,t]<- 0
E1    [   7   ,   4   ,i,t]<- 0
E1    [   7   ,   5   ,i,t]<- 0
E1    [   7   ,   6   ,i,t]<- 0
E1    [   7   ,   7   ,i,t]<- 1
E1    [   7   ,   8   ,i,t]<- 0
E1    [   7   ,   9   ,i,t]<- 0
E1    [   7   ,   10,i,t]<-   0
E1    [   7   ,   11,i,t]<-   0
E1    [   7   ,   12,i,t]<-   0

E1    [   8   ,   1   ,i,t]<- 0
E1    [   8   ,   2   ,i,t]<- 0
E1    [   8   ,   3   ,i,t]<- 0
E1    [   8   ,   4   ,i,t]<- 0
E1    [   8   ,   5   ,i,t]<- 0
E1    [   8   ,   6   ,i,t]<- 0
E1    [   8   ,   7   ,i,t]<- 0
E1    [   8   ,   8   ,i,t]<- 1
E1    [   8   ,   9   ,i,t]<- 0
E1    [   8   ,   10,i,t]<-   0
E1    [   8   ,   11,i,t]<-   0
E1    [   8   ,   12,i,t]<-   0

E1    [   9   ,   1   ,i,t]<- 0
E1    [   9   ,   2   ,i,t]<- 0
E1    [   9   ,   3   ,i,t]<- 0
E1    [   9   ,   4   ,i,t]<- 0
E1    [   9   ,   5   ,i,t]<- 0
E1    [   9   ,   6   ,i,t]<- 0
E1    [   9   ,   7   ,i,t]<- 0
E1    [   9   ,   8   ,i,t]<- 0
E1    [   9   ,   9   ,i,t]<- 1-alpha[i,t+1]
E1    [   9   ,   10,i,t]<-   0
E1    [   9   ,   11,i,t]<-   alpha[i,t+1]
E1    [   9   ,   12,i,t]<-   0

E1    [   10  ,   1,i,t]<-    0
E1    [   10  ,   2,i,t]<-    0
E1    [   10  ,   3,i,t]<-    0
E1    [   10  ,   4,i,t]<-    0
E1    [   10  ,   5,i,t]<-    0
E1    [   10  ,   6,i,t]<-    0
E1    [   10  ,   7,i,t]<-    0
E1    [   10  ,   8,i,t]<-    0
E1    [   10  ,   9,i,t]<-    2*(1-alpha[i,t+1])*alpha[i,t+1]
E1    [   10  ,   10,i,t]<-   1 - (2*(1-alpha[i,t+1])*alpha[i,t+1]) - (alpha[i,t+1])^2
E1    [   10  ,   11,i,t]<-   (alpha[i,t+1])^2
E1    [   10  ,   12,i,t]<-   0

E1    [   11  ,   1   ,i,t]<- 0
```

```
E1    [   11  ,   2   ,i,t]<- 0
E1    [   11  ,   3   ,i,t]<- 0
E1    [   11  ,   4   ,i,t]<- 0
E1    [   11  ,   5   ,i,t]<- 0
E1    [   11  ,   6   ,i,t]<- 0
E1    [   11  ,   7   ,i,t]<- 0
E1    [   11  ,   8   ,i,t]<- 0
E1    [   11  ,   9   ,i,t]<- 0
E1    [   11  ,   10,i,t]<-   0
E1    [   11  ,   11,i,t]<-   1
E1    [   11  ,   12,i,t]<-   0

E1    [   12  ,   1   ,i,t]<- 0
E1    [   12  ,   2   ,i,t]<- 0
E1    [   12  ,   3   ,i,t]<- 0
E1    [   12  ,   4   ,i,t]<- 0
E1    [   12  ,   5   ,i,t]<- 0
E1    [   12  ,   6   ,i,t]<- 0
E1    [   12  ,   7   ,i,t]<- 0
E1    [   12  ,   8   ,i,t]<- 0
E1    [   12  ,   9   ,i,t]<- 0
E1    [   12  ,   10,i,t]<-   0
E1    [   12  ,   11,i,t]<-   0
E1    [   12  ,   12,i,t]<-   1

# for recapture probability
E2    [   1   ,   1   ,i,t]<- 1 -p
E2    [   1   ,   2   ,i,t]<- p
E2    [   1   ,   3   ,i,t]<- 0
E2    [   1   ,   4   ,i,t]<- 0
E2    [   1   ,   5   ,i,t]<- 0
E2    [   1   ,   6   ,i,t]<- 0
E2    [   1   ,   7   ,i,t]<- 0
E2    [   1   ,   8   ,i,t]<- 0
E2    [   1   ,   9   ,i,t]<- 0
E2    [   1   ,   10,i,t]<-   0
E2    [   1   ,   11,i,t]<-   0
E2    [   1   ,   12,i,t]<-   0

E2    [   2   ,   1   ,i,t]<- 1 -p
E2    [   2   ,   2   ,i,t]<- 0
E2    [   2   ,   3   ,i,t]<- p
E2    [   2   ,   4   ,i,t]<- 0
E2    [   2   ,   5   ,i,t]<- 0
E2    [   2   ,   6   ,i,t]<- 0
E2    [   2   ,   7   ,i,t]<- 0
E2    [   2   ,   8   ,i,t]<- 0
E2    [   2   ,   9   ,i,t]<- 0
E2    [   2   ,   10,i,t]<-   0
E2    [   2   ,   11,i,t]<-   0
E2    [   2   ,   12,i,t]<-   0

E2    [   3   ,   1   ,i,t]<- 1 -p
```

```
    E2    [   3   ,   2   ,i,t]<- 0
    E2    [   3   ,   3   ,i,t]<- 0
    E2    [   3   ,   4   ,i,t]<- p
    E2    [   3   ,   5   ,i,t]<- 0
    E2    [   3   ,   6   ,i,t]<- 0
    E2    [   3   ,   7   ,i,t]<- 0
    E2    [   3   ,   8   ,i,t]<- 0
    E2    [   3   ,   9   ,i,t]<- 0
    E2    [   3   ,   10,i,t]<-   0
    E2    [   3   ,   11,i,t]<-   0
    E2    [   3   ,   12,i,t]<-   0

    E2    [   4   ,   1   ,i,t]<- 1 -p
    E2    [   4   ,   2   ,i,t]<- 0
    E2    [   4   ,   3   ,i,t]<- 0
    E2    [   4   ,   4   ,i,t]<- 0
    E2    [   4   ,   5   ,i,t]<- p
    E2    [   4   ,   6   ,i,t]<- 0
    E2    [   4   ,   7   ,i,t]<- 0
    E2    [   4   ,   8   ,i,t]<- 0
    E2    [   4   ,   9   ,i,t]<- 0
    E2    [   4   ,   10,i,t]<-   0
    E2    [   4   ,   11,i,t]<-   0
    E2    [   4   ,   12,i,t]<-   0

    E2    [   5   ,   1,i,t]<-    1 -p
    E2    [   5   ,   2,i,t]<-    0
    E2    [   5   ,   3,i,t]<-    0
    E2    [   5   ,   4,i,t]<-    0
    E2    [   5   ,   5,i,t]<-    0
    E2    [   5   ,   6,i,t]<-    p
    E2    [   5   ,   7,i,t]<-    0
    E2    [   5   ,   8,i,t]<-    0
    E2    [   5   ,   9,i,t]<-    0
    E2    [   5   ,   10,i,t]<-   0
    E2    [   5   ,   11,i,t]<-   0
    E2    [   5   ,   12,i,t]<-   0

    E2    [   6   ,   1   ,i,t]<- 1 -p
    E2    [   6   ,   2   ,i,t]<- 0
    E2    [   6   ,   3   ,i,t]<- 0
    E2    [   6   ,   4   ,i,t]<- 0
    E2    [   6   ,   5   ,i,t]<- 0
    E2    [   6   ,   6   ,i,t]<- 0
    E2    [   6   ,   7   ,i,t]<- p
    E2    [   6   ,   8   ,i,t]<- 0
    E2    [   6   ,   9   ,i,t]<- 0
    E2    [   6   ,   10,i,t]<-   0
    E2    [   6   ,   11,i,t]<-   0
    E2    [   6   ,   12,i,t]<-   0

    E2    [   7   ,   1   ,i,t]<- 1 -p
    E2    [   7   ,   2   ,i,t]<- 0
```

```
E2    [   7   ,   3   ,i,t]<- 0
E2    [   7   ,   4   ,i,t]<- 0
E2    [   7   ,   5   ,i,t]<- 0
E2    [   7   ,   6   ,i,t]<- 0
E2    [   7   ,   7   ,i,t]<- 0
E2    [   7   ,   8   ,i,t]<- p
E2    [   7   ,   9   ,i,t]<- 0
E2    [   7   ,   10,i,t]<-   0
E2    [   7   ,   11,i,t]<-   0
E2    [   7   ,   12,i,t]<-   0

E2    [   8   ,   1   ,i,t]<- 1 -p
E2    [   8   ,   2   ,i,t]<- 0
E2    [   8   ,   3   ,i,t]<- 0
E2    [   8   ,   4   ,i,t]<- 0
E2    [   8   ,   5   ,i,t]<- 0
E2    [   8   ,   6   ,i,t]<- 0
E2    [   8   ,   7   ,i,t]<- 0
E2    [   8   ,   8   ,i,t]<- 0
E2    [   8   ,   9   ,i,t]<- p
E2    [   8   ,   10,i,t]<-   0
E2    [   8   ,   11,i,t]<-   0
E2    [   8   ,   12,i,t]<-   0

E2    [   9   ,   1   ,i,t]<- 1-p
E2    [   9   ,   2   ,i,t]<- 0
E2    [   9   ,   3   ,i,t]<- 0
E2    [   9   ,   4   ,i,t]<- 0
E2    [   9   ,   5   ,i,t]<- 0
E2    [   9   ,   6   ,i,t]<- 0
E2    [   9   ,   7   ,i,t]<- 0
E2    [   9   ,   8   ,i,t]<- 0
E2    [   9   ,   9   ,i,t]<- 0
E2    [   9   ,   10,i,t]<-   p
E2    [   9   ,   11,i,t]<-   0
E2    [   9   ,   12,i,t]<-   0

E2    [   10  ,   1,i,t]<-    1-p
E2    [   10  ,   2,i,t]<-    0
E2    [   10  ,   3,i,t]<-    0
E2    [   10  ,   4,i,t]<-    0
E2    [   10  ,   5,i,t]<-    0
E2    [   10  ,   6,i,t]<-    0
E2    [   10  ,   7,i,t]<-    0
E2    [   10  ,   8,i,t]<-    0
E2    [   10  ,   9,i,t]<-    0
E2    [   10  ,   10,i,t]<-   0
E2    [   10  ,   11,i,t]<-   p
E2    [   10  ,   12,i,t]<-   0

E2    [   11  ,   1   ,i,t]<- 1 - p
E2    [   11  ,   2   ,i,t]<- 0
E2    [   11  ,   3   ,i,t]<- 0
```

```
    E2    [   11  ,   4   ,i,t]<- 0
    E2    [   11  ,   5   ,i,t]<- 0
    E2    [   11  ,   6   ,i,t]<- 0
    E2    [   11  ,   7   ,i,t]<- 0
    E2    [   11  ,   8   ,i,t]<- 0
    E2    [   11  ,   9   ,i,t]<- 0
    E2    [   11  ,   10,i,t]<-   0
    E2    [   11  ,   11,i,t]<-   0
    E2    [   11  ,   12,i,t]<-   p

    E2    [   12  ,   1   ,i,t]<- 1
    E2    [   12  ,   2   ,i,t]<- 0
    E2    [   12  ,   3   ,i,t]<- 0
    E2    [   12  ,   4   ,i,t]<- 0
    E2    [   12  ,   5   ,i,t]<- 0
    E2    [   12  ,   6   ,i,t]<- 0
    E2    [   12  ,   7   ,i,t]<- 0
    E2    [   12  ,   8   ,i,t]<- 0
    E2    [   12  ,   9   ,i,t]<- 0
    E2    [   12  ,   10,i,t]<-   0
    E2    [   12  ,   11,i,t]<-   0
    E2    [   12  ,   12,i,t]<-   0

    # Matrix product for offspring independence and recapture
    E[1:12,1:12,i,t] <- E1[1:12,1:12,i,t] %*% E2[1:12,1:12,i,t]
  }
}

## LIKELIHOOD

for (i in 1:N)  # for each individual
{
  # The estimated probabilities of initial states S0 are the proportions in each state
  #at first capture occasion
  alive[i,First[i]] ~ dcat(S0[1:12])
  mydata[i,First[i]] ~ dcat(E0[alive[i,First[i]],1:12])

  for (j in (First[i]+1):Years)
  {

    ## STATE EQUATIONS ##
    # draw S(t) given S(t-1)
    alive[i,j] ~ dcat(S[alive[i,j-1],1:12])

    ## OBSERVATION EQUATIONS ##
    # draw events E(t) given states S(t)

    mydata[i,j] ~ dcat(E[alive[i,j],1:12,i,j-1])

  }
}
```

```
  ## PRIORS
  # capture probability
  p ~ dunif(0,1)

  # juveniles, subadults and adult survival
  phi ~ dunif(0,1)

  # initial states
  for (i in 1:10){ log(prop[i]) <- theta[i]
  theta[i] ~ dnorm(0,1)}

  # offspring survival
  # litter survival n=2 offspring
  l02 <- 1 -(1- s[2]^2 -2*s[2]*(1-s[2]))
  l12 <- 1- (1- s[4]^2 -2*s[4]*(1-s[4]))
  # indiviual offspring survival
  for(i in 1:2){s[i]~ dunif(0,1)}
    # Set constraints
    for(u in 1:2){  X[u] ~ dunif(0,1)} # with X ~ U[0,1] then  (a + ( b - a ) * X)
    #so that s[1] < s[3] <phi[1] for litter of 1
    s[3] <- s[1] + (phi[1] - s[1]) * X[1]
    # and s[2] < s[4] < phi[1] for litters of 2
    s[4] <- s[2] + (phi[1] - s[2]) * X[2]

  # Breeding probability
  kappa ~ dunif(0,1)
  for(i in 1:4){beta[i]~ dunif(0,1)}

  # Litter size probability
  for(i in 1:4){gamma[i]~ dunif(0,1)}

} # end model

    ",fill=TRUE)
sink()
#######
#######
```

We used non-informative priors on the model parameters, with uniform distribution between 0 and 1 for probabilities, and normal distribution with mean 0 and variance of 1 for regression coefficients. To help estimation of the parameters in the model, we introduced the constraint that survival of cubs (age <1) was lower than that of yearling survival (aged 1). We used one chain with 10 000 iterations and 4000 burnin.

Load the data (family unit CR histories, initial state matrix, date of captures) and fit the model :

```
RES <- list()

for(r in 1:100){

nameCH <- paste(repname,"/simCH",r,"_p",p,"_T",n.occasions,".txt",sep="")
# load capture histories
data <- read.table(paste(nameCH),sep=" ",header=FALSE)
# initial values for state matrix
nameINIT <- paste(repname,"/siminit",r,"_p",p,"_T",n.occasions,".txt",sep="")
initmat <- read.table(paste(nameINIT),sep="")
```

```
# date of capture
nameDAY <- paste(repname,"/simdaycapt",r,"_p",p,"_T",n.occasions,".txt",sep="")
daycapt <- read.table(paste(nameDAY),sep="")

data <- data.matrix(data)
alive1 <- data.matrix(initmat)
head(data)
N <- dim(data)[1]
Years <- dim(data)[2]

# Compute vector with occasion of first capture
get.first <- function(x) min(which(x!=0))
First <- apply(data, 1, get.first)

# Predict departure probability function of date of capture
alpha = matrix(0,nrow=N,ncol=Years)
  for(i in 1:N){
    for(j in (First[i]+1):Years){
    alpha[i,j] <- predict(modeld,newdata=list(doy=daycapt[i,j]),type="response")
  }
}

# Bundle data for jags
mydatax <- list(N=N,First=First,Years=Years,mydata=data.matrix(data+1)
                ,alpha=data.matrix(alpha))

# Initial values
init1 <- list(theta=rnorm(10, mean = 0, sd = 1), alive=alive1) #
inits <- list(init1)

# Parameters monitored
params <- c("phi","kappa","s","l02","l12","beta","gamma","p","S0")

# Call JAGS from R to fit the model
out <- jags(data=mydatax,inits=inits, parameters.to.save=params,
            model.file = 'Multieventmodel_Fit_simul.txt',n.chains=1,
            n.iter=10000,n.burnin=4000)

RES[[r]] <- out # store results
print(paste(r))
}
save(RES,file=paste('RES',n.occasions,'p',p,'.RData',sep="")) # save results
```

Post process simulation results

Load and plot the results:

```
# load results of the simulations
capture <- c(0.25,0.25,0.5,0.5)
filenames <- c("S1RESp0.25alpha05.RData","S2RESp0.25alphaDate.RData",
               "S3RESp0.5alpha05.RData","S4RESp0.5alphaDate.RData")

EST <- CI1 <- CI2 <- simres <- biasall <- rMSEall <- list()
```

```
for(S in 1:4){
  p = capture[S]

load(paste(filenames[S]))

# parameters used to simulate the data in same order
# phi, kappa, s1,s2,s3,s4,beta1,beta2,beta3,beta4,gamma1,gamma2,gamma3,gamma4,p
theta <- c(0.9,0,0.6,0.55,0.8,0.75,0.5,0.7,0.9,0.8,0.4,0.5,0.6,0.7,as.numeric(paste(p)))
nparam <- length(theta)
nrepet <- length(RES)

# Calculate summary of posterior distribution estimated for all parameters
#(mean, and 95% credible interval) and calculate bias and root mean-square-errors :
  est <- ci1 <- ci2 <- BIAS <- pBIAS <- rMSE <- matrix(NA,nrow=length(RES),ncol=nparam)
  for(i in 1:nrepet){
    #get estimates
    est[i,] <- unlist(RES[[i]]$mean[c(1,2,3,6,7,8)])
    ci1[i,] <- unlist(RES[[i]]$q2.5[c(1,2,3,6,7,8)])
    ci2[i,] <- unlist(RES[[i]]$q97.5[c(1,2,3,6,7,8)])
    # absolute bias, and root mean square error
    BIAS[i,] <- (est[i,] - theta)
    rMSE[i,] <- sqrt( (est[i,] - theta)^2 )
  }#nrepet

# Plot the results
#    pdf(width=9,height=6,pointsize=4,file="test.pdf",paper="a4r")
    par(mfrow=c(3,5))
    xlabs <- c(expression(phi),expression(kappa),expression(S[1]),expression(S[2]),
               expression(S[3]),expression(S[4]),
               expression(beta[1]),expression(beta[2]),
               expression(beta[3]),expression(beta[4]),
               expression(gamma[1]),expression(gamma[2]),
               expression(gamma[3]),expression(gamma[4]),
               expression(p))

    for(j in 1:nparam){
      plot(est[,j], 1:nrepet, ylab='',xlim=c(0,1),las=TRUE,cex=1,type="n",
           main=xlabs[j],xlab=paste("bias = ",round(mean(BIAS[,j]),3),
                                    "; rmse =",round(mean(rMSE[,j]),2), sep=""))
      segments(ci1[,j], 1:100,ci2[,j], 1:nrepet,col="grey70",lwd=0.5)
   points(est[,j], 1:nrepet,col="black",cex=0.4,pch=16,)
   abline(v=theta[j], lty=2, col='red')
   }
#    dev.off()

  #store results
  EST[[S]] <- est
  CI1[[S]] <- ci1
  CI2[[S]] <- ci2
  simres[[S]] <- RES
  biasall[[S]] <- BIAS
  rMSEall[[S]] <- rMSE
```

```
} #S
```

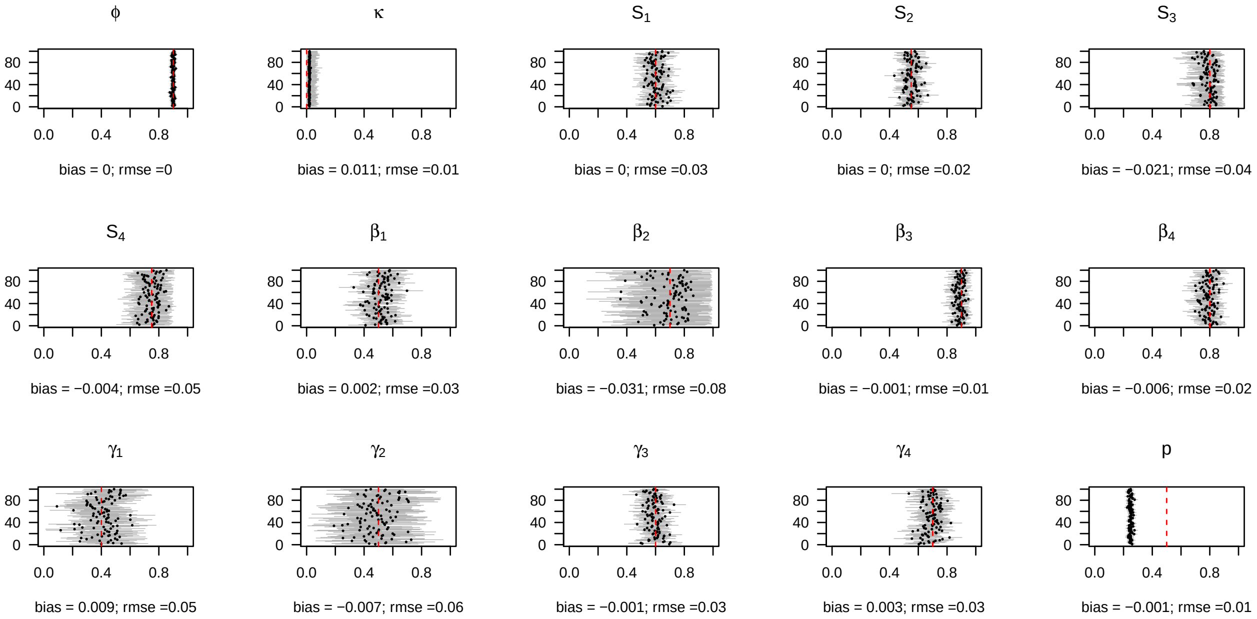

Figure S3. Performance of the model on simulated data with low detection with constant departure (scenario S1). For each of the 100 simulated data sets, we displayed the mean (circle) and the 95% confidence interval (horizontal solid line) of the parameter. The actual value of the parameter is given by the vertical dashed red line. The estimated absolute bias and root-mean-square error are provided in the legend of the X-axis for each parameter.

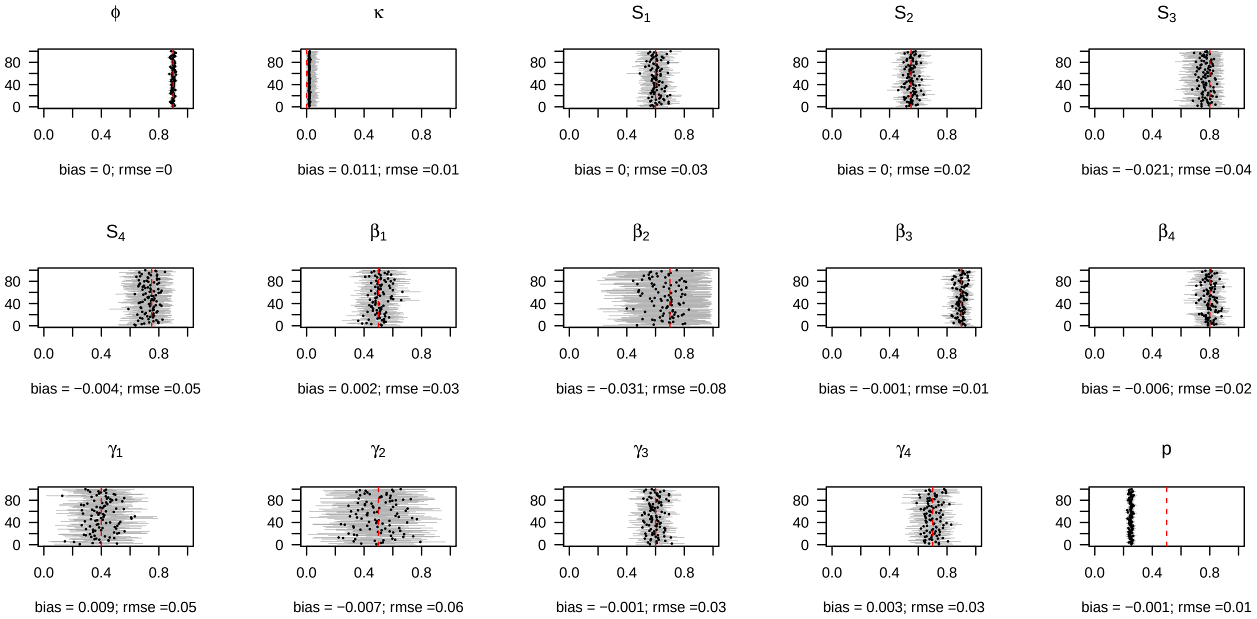

Figure S4. Performance of the model on simulated data with low detection with departure varying with date of capture (scenario S2). For each of the 100 simulated data sets, we displayed the mean (circle) and the 95% confidence interval (horizontal solid line) of the parameter. The actual value of the parameter is given by the vertical dashed red line. The estimated absolute bias and root-mean-square error are provided in the legend of the X-axis for each parameter.

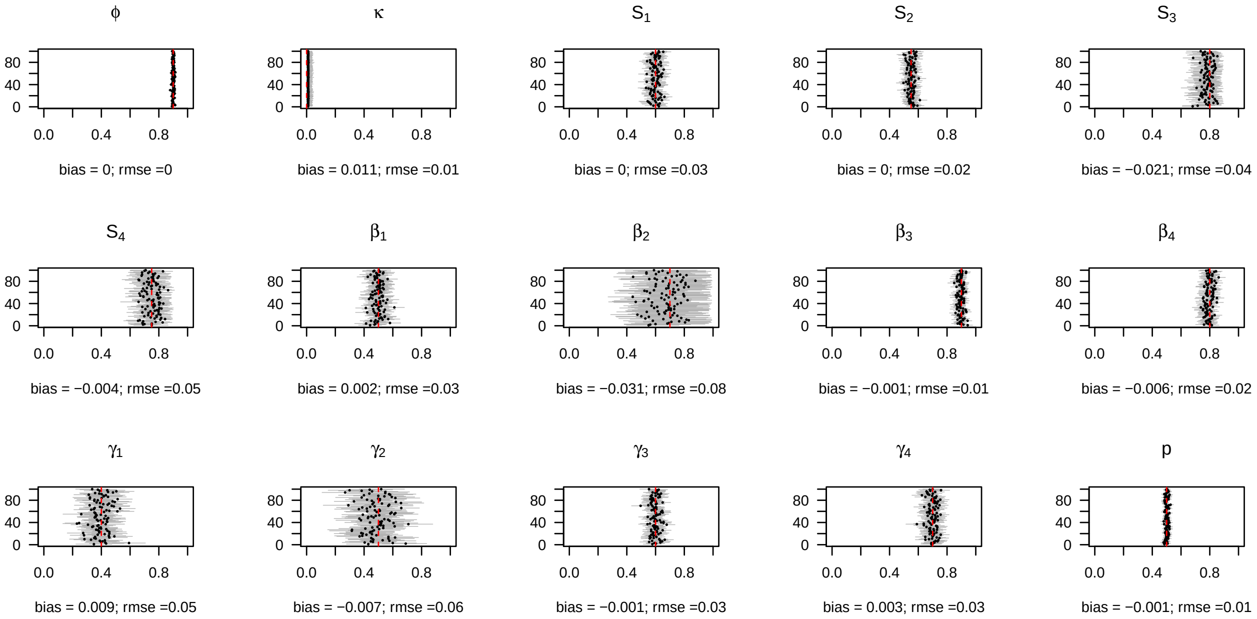

Figure S5. Performance of the model on simulated data with high detection with constant departure (scenario S3). For each of the 100 simulated data sets, we displayed the mean (circle) and the 95% confidence interval (horizontal solid line) of the parameter. The actual value of the parameter is given by the vertical dashed red line. The estimated absolute bias and root-mean-square error are provided in the legend of the X-axis for each parameter.

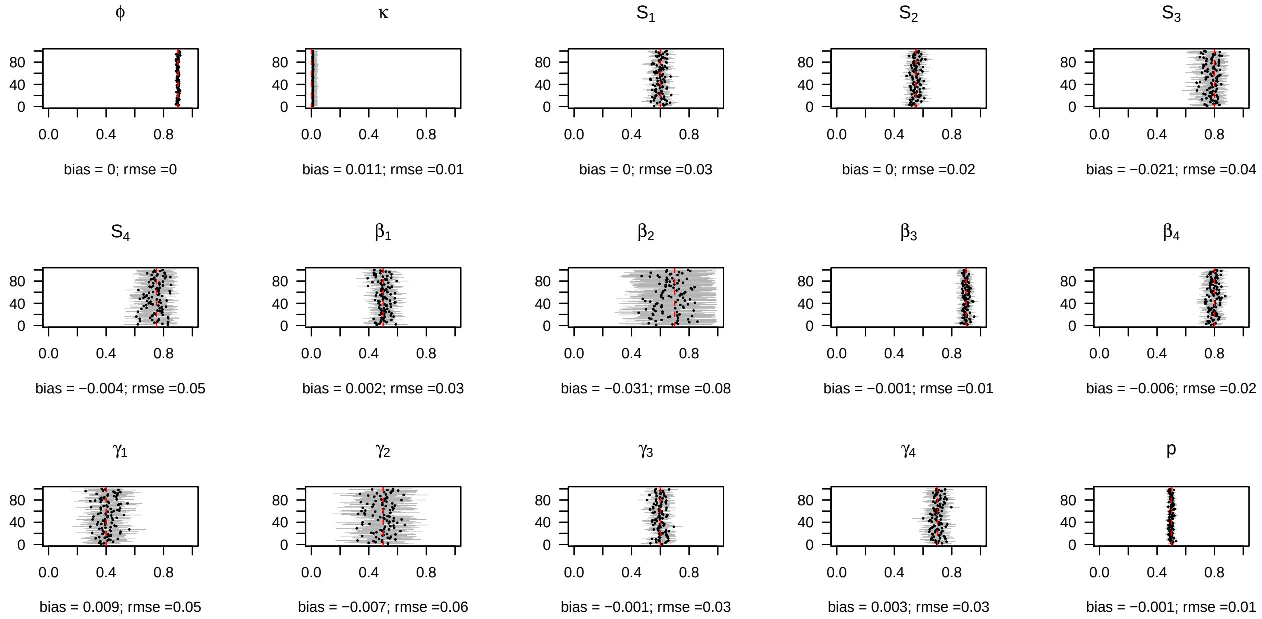

Figure S6. Performance of the model on simulated data with high detection with departure varying with date of capture (scenario S4). For each of the 100 simulated data sets, we displayed the mean (circle) and the 95% confidence interval (horizontal solid line) of the parameter. The actual value of the parameter is given by the vertical dashed red line. The estimated absolute bias and root-mean-square error are provided in the legend of the X-axis for each parameter.

Make tables with values of bias and rsme for each parameter for all scenarios:

```
# average bias
mean(c(biasall[[1]],biasall[[2]],biasall[[3]],biasall[[4]]))
```

```
## [1] -0.002881172
```

```
# average rmse
mean(c(rMSEall[[1]],rMSEall[[2]],rMSEall[[3]],rMSEall[[4]]))
```

```
## [1] 0.03638131
```

```
tabB <- rbind( round(apply(biasall[[1]],2,mean),2), round(apply(biasall[[2]],2,mean),2),
       round(apply(biasall[[3]],2,mean),2), round(apply(biasall[[4]],2,mean),2) )

tabR <- rbind( round(apply(rMSEall[[1]],2,mean),2), round(apply(rMSEall[[2]],2,mean),2),
       round(apply(rMSEall[[3]],2,mean),2), round(apply(rMSEall[[4]],2,mean),2) )

parnames <- c(expression(phi),expression(kappa),expression(S[1]),expression(S[2]),
              expression(S[3]),expression(S[4]),
               expression(beta[1]),expression(beta[2]),expression(beta[3]),
              expression(beta[4]),expression(gamma[1]),expression(gamma[2]),expression(gamma[3]),
              expression(gamma[4]), expression(p))
colnames(tabB) <- colnames(tabR) <- parnames
rownames(tabB) <- rownames(tabR) <- c("S1","S2","S3","S4")

tabB
```

```
##    phi kappa S[1] S[2]  S[3]  S[4] beta[1] beta[2] beta[3] beta[4] gamma[1]
## S1   0  0.02 0.00    0 -0.01  0.00    0.02   -0.01   -0.01   -0.01     0.01
## S2   0  0.02 0.01    0 -0.03 -0.02    0.01   -0.04    0.00    0.00     0.00
```

```
## S3    0  0.01 0.00     0 -0.01  0.00     0.00    -0.03     0.00     0.00      0.00
## S4    0  0.01 0.00     0 -0.02  0.00     0.00    -0.03     0.00    -0.01      0.01
##     gamma[2] gamma[3] gamma[4] p
## S1    -0.01    -0.01    -0.01 0
## S2     0.00     0.00     0.00 0
## S3    -0.01     0.00     0.00 0
## S4    -0.01     0.00     0.00 0
```

```
tabR
```

```
##      phi kappa S[1] S[2] S[3] S[4] beta[1] beta[2] beta[3] beta[4] gamma[1]
## S1 0.01  0.02 0.04 0.03 0.04 0.04    0.05    0.09    0.02    0.03     0.08
## S2 0.01  0.02 0.03 0.03 0.04 0.04    0.04    0.09    0.02    0.03     0.07
## S3 0.00  0.01 0.02 0.02 0.03 0.04    0.03    0.08    0.02    0.02     0.05
## S4 0.00  0.01 0.03 0.02 0.04 0.05    0.03    0.08    0.01    0.02     0.05
##     gamma[2] gamma[3] gamma[4]    p
## S1     0.09     0.04     0.04 0.01
## S2     0.11     0.04     0.04 0.01
## S3     0.07     0.02     0.02 0.01
## S4     0.06     0.03     0.03 0.01
```

```
#write.csv(tabB,file = "RES_biasperparam.csv")
#write.csv(tabR,file = "RES_rmseperparam.csv")
```

Bibliogrpahy

Kéry, M., & Schaub, M. (2011). Bayesian population analysis using WinBUGS: a hierarchical perspective. Academic Press.
