# Supplementary material for: Modeling the demography of species providing extended parental care: A capture–recapture multievent model with a case study on polar bears (Ursus maritimus)
Source: Ecol Evol. 2021 Mar 10;11(7):3380–92. doi: 10.1002/ece3.7296 (PMC8019049; doi:10.1002/ece3.7296)
Supplement: Supplementary file 2 — Appendix S2 [file ECE3-11-3380-s002.pdf]

Guidance to fit the model to the polar bear data, R code, and additional results.

Sarah Cubaynes

6/23/2020

We fitted our model to the Polar bear data in a Bayesian framework using Markov Chain Monte Carlo (MCMC) simulation implemented in program JAGS (Hornik et al. 2003) called from R using package jagsUI (Kellner 2015). We ran two MCMC in parallel with different initial values, we used 20.000 iterations with an initial burn-in of 9.000 iterations, thinning every 5 iterations, to reach convergence to a stationary distribution, assessed by visual inspection of trace plots for each model parameter to ensure adequate mixing and by using the Gelman and Rubin diagnostic (R-hat < 1.02). We used non-informative priors on the model parameters, with uniform distribution between 0 and 1 for probabilities, and normal distribution with mean 0 and variance of 1 for regression coefficients. To help estimation of the parameters in the model, we introduced the constraint that survival of cubs was lower than that of yearling survival (Amstrup and Durner, 1995). The constraint was enough to reach convergence with satisfactory posterior distribution for each of the estimated parameters (see Figure below).

Below we provide the code to prepare the data, run the model and analyse the results. All files associated with Appendix 2, including the polar bear data files, are available on GitHub here.

Prepare data and run jags model

Load data and useful packages :

```
load(file = "CRlocalbears_revision2MEE.Rdata") # family units CR histories
data <- data.matrix(CRlb)

load(file = "daylocalbears_revision2MEE.Rdata") # capture date in day of the yaer
daycapt <- daylb
load(file="initstatelocalbears_revision2MEE.Rdata")#matrix of initial states

alive1 <- data.matrix(initmatlb)

load(file = "dataweaning_revision2MEE.Rdata") # all two-year old bear captures

library(jagsUI) # to run jags model
```

```
## Loading required package: lattice

##
## Attaching package: 'jagsUI'

## The following object is masked from 'package:utils':
##
##     View
```

```r
library(jtools) # to make predict plot from glm
```

Define useful quantities:

```r
N <- dim(data)[1] # number of family units
Years <- dim(data)[2] #number of sampling occasions

# Compute vector with occasion of first capture
get.first <- function(x) min(which(x!=0))
First <- apply(data, 1, get.first)
```

We use the ratio of two-year old bears captured alone versus still together with their mother (include all bears, not only resident females) to estimate the shape of the relationship between offspring departure probability and date within the field season:

```r
nty <- dim(dataweaning)[[1]] # number of two-year old bears captured
status <- dataweaning$status #status of two-year old bears at the time of capture :
#1 alone (already departed from family unit),
#0 still together with mother (not yet departed from family unit)
doy <- dataweaning$daysinseason # date of capture

# glm of departure probability as a function of date of capture
modeld<-glm(status~doy,family="binomial")
summary(modeld)
```

```
##
## Call:
## glm(formula = status ~ doy, family = "binomial")
##
## Deviance Residuals:
##     Min       1Q   Median       3Q      Max
## -1.7056  -1.2304   0.7665   0.9143   1.3215
##
## Coefficients:
##             Estimate Std. Error z value Pr(>|z|)
## (Intercept) -3.56486    1.75383  -2.033   0.0421 *
## doy          0.03803    0.01646   2.310   0.0209 *
## ---
## Signif. codes:  0 '***' 0.001 '**' 0.01 '*' 0.05 '.' 0.1 ' ' 1
##
## (Dispersion parameter for binomial family taken to be 1)
##
##     Null deviance: 154.11  on 119  degrees of freedom
## Residual deviance: 137.99  on 118  degrees of freedom
## AIC: 141.99
##
## Number of Fisher Scoring iterations: 6
```

Plot predicted departure probability of two-year old bears as a function of date in day of the year (doy):

```r
effect_plot(modeld, pred = doy, pred.values=80:130,interval = TRUE,
            plot.points = FALSE,y.label = "departure probability")
```

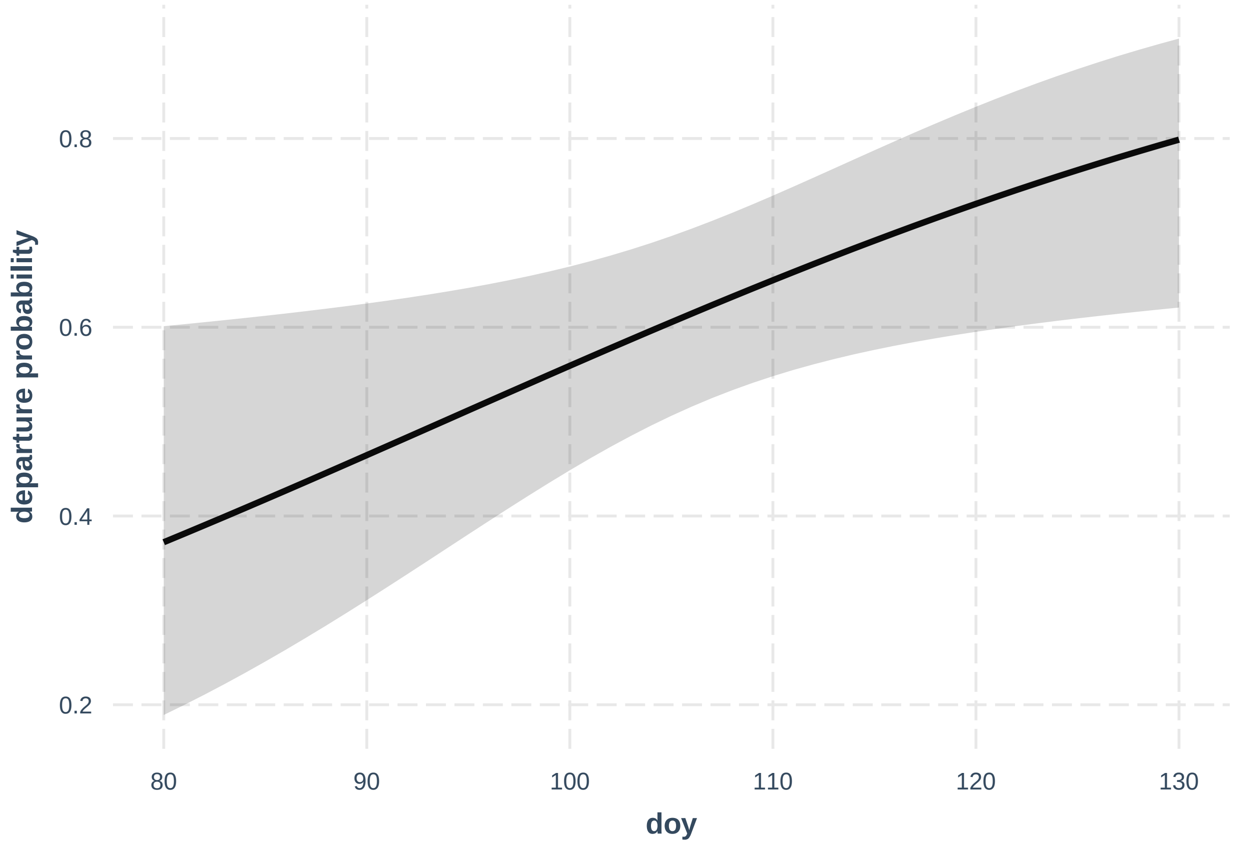

Departure probability increased throughout the field season. It was about 40% at the end of March and reached 80% at mid-May.

Predict departure probability from date of capture for resident family units (departure probability will be used in matrix E2 of the observation process to relate state to events for family units in states AS1 or AS2):

```
# Predict departure probability function of date of capture
daycapt <- daylb # dates of capture

# if not captured, replace with 80 (in date of capture) to avoid NAs
daycapt <- replace(daycapt,is.na(daycapt),80)

# create empty object to store predicted departure probability
alpha = matrix(0,nrow=N,ncol=Years)
# predict departure probability based on date of capture using modeld
for(i in 1:N){
  for(j in (First[i]):Years){
    alpha[i,j] <- predict(modeld,newdata=list(doy=daycapt[i,j]),type="response")
  }
}
```

Create a list containing the data to run the jags model:

```
# Bundle data for jags
mydatax <- list(N=N,First=First,Years=Years,mydata=data.matrix(data+1),alpha=data.matrix(alpha))
```

Generate initial values for each of the two chains:

```
set.seed(42)
# Initial values
```

```r
init1 <- list(theta=rnorm(10, mean = 0, sd = 1),alive=alive1)
init2 <- list(theta=rnorm(10, mean = 0, sd = 1),alive=alive1)
inits <- list(init1,init2)
```

Create a list with names of the parameters to monitor:

```r
# Parameters monitored
params <- c("phi","s","l02","l12","kappa","beta","gamma","p","prop")
```

Load the script of the jags model :

```r
# JAGS MODEL
sink("Multieventmodel_FitresidentBeardata.txt")
cat("

model {
  # Probabilities of events given states and states given states

  # vector of initial states
  S0[1] <- prop[1] / (1 + sum(prop[1:10])) # prob. of being in initial state J2
  S0[2] <- prop[2] / (1 + sum(prop[1:10])) # prob. of being in initial state J3
  S0[3] <- prop[3] / (1 + sum(prop[1:10])) # prob. of being in initial state SA4
  S0[4] <- prop[4] / (1 + sum(prop[1:10])) # prob. of being in initial state SA5
  S0[5] <- prop[5] / (1 + sum(prop[1:10])) # prob. of being in initial state A01
  S0[6] <- prop[6] / (1 + sum(prop[1:10])) # prob. of being in initial state A02
  S0[7] <- prop[7] / (1 + sum(prop[1:10])) # prob. of being in initial state A11
  S0[8] <- prop[8] / (1 + sum(prop[1:10])) # prob. of being in initial state A12
  S0[9] <- prop[9] / (1 + sum(prop[1:10])) # prob. of being in initial state AS1
  S0[10] <- prop[10] / (1 + sum(prop[1:10])) # prob. of being in initial state AS2
  S0[11] <- 1 / (1 + sum(prop[1:10])) # prob. of being in initial state A-
  S0[12] <- 0 # prob. of being in initial state dead

  # State process: define probabilities of S(t+1) given S(t)
  # define PHI matrix gathering survival of independent juveniles, subadults and adults
  PHI   [   1   ,   1   ]<- phi[1]
  PHI   [   1   ,   2   ]<- 0
  PHI   [   1   ,   3   ]<- 0
  PHI   [   1   ,   4   ]<- 0
  PHI   [   1   ,   5   ]<- 0
  PHI   [   1   ,   6   ]<- 0
  PHI   [   1   ,   7   ]<- 0
  PHI   [   1   ,   8   ]<- 0
  PHI   [   1   ,   9   ]<- 0
  PHI   [   1   ,   10  ]<- 0
  PHI   [   1   ,   11  ]<- 0
  PHI   [   1   ,   12  ]<- 1-phi[1]

  PHI   [   2   ,   1   ]<- 0
  PHI   [   2   ,   2   ]<- phi[1]
  PHI   [   2   ,   3   ]<- 0
  PHI   [   2   ,   4   ]<- 0
  PHI   [   2   ,   5   ]<- 0
  PHI   [   2   ,   6   ]<- 0
  PHI   [   2   ,   7   ]<- 0
  PHI   [   2   ,   8   ]<- 0
```

```
PHI   [   2   ,   9   ]<- 0
PHI   [   2   ,   10  ]<- 0
PHI   [   2   ,   11  ]<- 0
PHI   [   2   ,   12  ]<- 1-phi[1]

PHI   [   3   ,   1   ]<- 0
PHI   [   3   ,   2   ]<- 0
PHI   [   3   ,   3   ]<- phi[1]
PHI   [   3   ,   4   ]<- 0
PHI   [   3   ,   5   ]<- 0
PHI   [   3   ,   6   ]<- 0
PHI   [   3   ,   7   ]<- 0
PHI   [   3   ,   8   ]<- 0
PHI   [   3   ,   9   ]<- 0
PHI   [   3   ,   10  ]<- 0
PHI   [   3   ,   11  ]<- 0
PHI   [   3   ,   12  ]<- 1-phi[1]

PHI   [   4   ,   1   ]<- 0
PHI   [   4   ,   2   ]<- 0
PHI   [   4   ,   3   ]<- 0
PHI   [   4   ,   4   ]<- phi[1]
PHI   [   4   ,   5   ]<- 0
PHI   [   4   ,   6   ]<- 0
PHI   [   4   ,   7   ]<- 0
PHI   [   4   ,   8   ]<- 0
PHI   [   4   ,   9   ]<- 0
PHI   [   4   ,   10  ]<- 0
PHI   [   4   ,   11  ]<- 0
PHI   [   4   ,   12  ]<- 1 - phi[1]

PHI   [   5   ,   1   ]<- 0
PHI   [   5   ,   2   ]<- 0
PHI   [   5   ,   3   ]<- 0
PHI   [   5   ,   4   ]<- 0
PHI   [   5   ,   5   ]<- phi[1]
PHI   [   5   ,   6   ]<- 0
PHI   [   5   ,   7   ]<- 0
PHI   [   5   ,   8   ]<- 0
PHI   [   5   ,   9   ]<- 0
PHI   [   5   ,   10  ]<- 0
PHI   [   5   ,   11  ]<- 0
PHI   [   5   ,   12  ]<- 1 - phi[1]

PHI   [   6   ,   1   ]<- 0
PHI   [   6   ,   2   ]<- 0
PHI   [   6   ,   3   ]<- 0
PHI   [   6   ,   4   ]<- 0
PHI   [   6   ,   5   ]<- 0
PHI   [   6   ,   6   ]<- phi[1]
PHI   [   6   ,   7   ]<- 0
PHI   [   6   ,   8   ]<- 0
PHI   [   6   ,   9   ]<- 0
```

```
PHI   [   6   ,   10  ]<- 0
PHI   [   6   ,   11  ]<- 0
PHI   [   6   ,   12  ]<- 1 - phi[1]

PHI   [   7   ,   1   ]<- 0
PHI   [   7   ,   2   ]<- 0
PHI   [   7   ,   3   ]<- 0
PHI   [   7   ,   4   ]<- 0
PHI   [   7   ,   5   ]<- 0
PHI   [   7   ,   6   ]<- 0
PHI   [   7   ,   7   ]<- phi[1]
PHI   [   7   ,   8   ]<- 0
PHI   [   7   ,   9   ]<- 0
PHI   [   7   ,   10  ]<- 0
PHI   [   7   ,   11  ]<- 0
PHI   [   7   ,   12  ]<- 1-phi[1]

PHI   [   8   ,   1   ]<- 0
PHI   [   8   ,   2   ]<- 0
PHI   [   8   ,   3   ]<- 0
PHI   [   8   ,   4   ]<- 0
PHI   [   8   ,   5   ]<- 0
PHI   [   8   ,   6   ]<- 0
PHI   [   8   ,   7   ]<- 0
PHI   [   8   ,   8   ]<- phi[1]
PHI   [   8   ,   9   ]<- 0
PHI   [   8   ,   10  ]<- 0
PHI   [   8   ,   11  ]<- 0
PHI   [   8   ,   12  ]<- 1-phi[1]

PHI   [   9   ,   1   ]<- 0
PHI   [   9   ,   2   ]<- 0
PHI   [   9   ,   3   ]<- 0
PHI   [   9   ,   4   ]<- 0
PHI   [   9   ,   5   ]<- 0
PHI   [   9   ,   6   ]<- 0
PHI   [   9   ,   7   ]<- 0
PHI   [   9   ,   8   ]<- 0
PHI   [   9   ,   9   ]<- phi[1]
PHI   [   9   ,   10  ]<- 0
PHI   [   9   ,   11  ]<- 0
PHI   [   9   ,   12  ]<- 1-phi[1]

PHI   [   10  ,   1   ]<- 0
PHI   [   10  ,   2   ]<- 0
PHI   [   10  ,   3   ]<- 0
PHI   [   10  ,   4   ]<- 0
PHI   [   10  ,   5   ]<- 0
PHI   [   10  ,   6   ]<- 0
PHI   [   10  ,   7   ]<- 0
PHI   [   10  ,   8   ]<- 0
PHI   [   10  ,   9   ]<- 0
PHI   [   10  ,   10  ]<- phi[1]
```

```
PHI   [   10  ,   11  ]<- 0
PHI   [   10  ,   12  ]<- 1-phi[1]

PHI   [   11  ,   1   ]<- 0
PHI   [   11  ,   2   ]<- 0
PHI   [   11  ,   3   ]<- 0
PHI   [   11  ,   4   ]<- 0
PHI   [   11  ,   5   ]<- 0
PHI   [   11  ,   6   ]<- 0
PHI   [   11  ,   7   ]<- 0
PHI   [   11  ,   8   ]<- 0
PHI   [   11  ,   9   ]<- 0
PHI   [   11  ,   10  ]<- 0
PHI   [   11  ,   11  ]<- phi[1]
PHI   [   11  ,   12  ]<- 1-phi[1]

PHI   [   12  ,   1   ]<- 0
PHI   [   12  ,   2   ]<- 0
PHI   [   12  ,   3   ]<- 0
PHI   [   12  ,   4   ]<- 0
PHI   [   12  ,   5   ]<- 0
PHI   [   12  ,   6   ]<- 0
PHI   [   12  ,   7   ]<- 0
PHI   [   12  ,   8   ]<- 0
PHI   [   12  ,   9   ]<- 0
PHI   [   12  ,   10  ]<- 0
PHI   [   12  ,   11  ]<- 0
PHI   [   12  ,   12  ]<- 1

# Define PSI matrices gathering state-to-state transition probabilities, it includes:
# PSI1: offspring survival and growth to next age, proba of sexual maturation:
PSI1  [   1   ,   1   ]<- 1
PSI1  [   1   ,   2   ]<- 0
PSI1  [   1   ,   3   ]<- 0
PSI1  [   1   ,   4   ]<- 0
PSI1  [   1   ,   5   ]<- 0
PSI1  [   1   ,   6   ]<- 0
PSI1  [   1   ,   7   ]<- 0
PSI1  [   1   ,   8   ]<- 0
PSI1  [   1   ,   9   ]<- 0
PSI1  [   1   ,   10  ]<- 0
PSI1  [   1   ,   11  ]<- 0
PSI1  [   1   ,   12  ]<- 0
PSI1  [   1   ,   13  ]<- 0

PSI1  [   2   ,   1   ]<- 0
PSI1  [   2   ,   2   ]<- 1
PSI1  [   2   ,   3   ]<- 0
PSI1  [   2   ,   4   ]<- 0
PSI1  [   2   ,   5   ]<- 0
PSI1  [   2   ,   6   ]<- 0
PSI1  [   2   ,   7   ]<- 0
PSI1  [   2   ,   8   ]<- 0
```

```
PSI1  [   2   ,   9   ]<- 0
PSI1  [   2   ,   10  ]<- 0
PSI1  [   2   ,   11  ]<- 0
PSI1  [   2   ,   12  ]<- 0
PSI1  [   2   ,   13  ]<- 0

PSI1  [   3   ,   1   ]<- 0
PSI1  [   3   ,   2   ]<- 0
PSI1  [   3   ,   3   ]<- 1-kappa#1 #
PSI1  [   3   ,   4   ]<- 0
PSI1  [   3   ,   5   ]<- 0
PSI1  [   3   ,   6   ]<- 0
PSI1  [   3   ,   7   ]<- 0
PSI1  [   3   ,   8   ]<- 0
PSI1  [   3   ,   9   ]<- 0
PSI1  [   3   ,   10  ]<- 0
PSI1  [   3   ,   11  ]<- 0
PSI1  [   3   ,   12  ]<- kappa #0
PSI1  [   3   ,   13  ]<- 0

PSI1  [   4   ,   1   ]<- 0
PSI1  [   4   ,   2   ]<- 0
PSI1  [   4   ,   3   ]<- 0
PSI1  [   4   ,   4   ]<- 0
PSI1  [   4   ,   5   ]<- 0
PSI1  [   4   ,   6   ]<- 0
PSI1  [   4   ,   7   ]<- 0
PSI1  [   4   ,   8   ]<- 0
PSI1  [   4   ,   9   ]<- 0
PSI1  [   4   ,   10  ]<- 0
PSI1  [   4   ,   11  ]<- 0
PSI1  [   4   ,   12  ]<- 1
PSI1  [   4   ,   13  ]<- 0

PSI1  [   5   ,   1   ]<- 0
PSI1  [   5   ,   2   ]<- 0
PSI1  [   5   ,   3   ]<- 0
PSI1  [   5   ,   4   ]<- s[1] # litter of 1, cub survives
PSI1  [   5   ,   5   ]<- 0
PSI1  [   5   ,   6   ]<- 0
PSI1  [   5   ,   7   ]<- 0
PSI1  [   5   ,   8   ]<- 1-s[1] #litter of 1, cub dies
PSI1  [   5   ,   9   ]<- 0
PSI1  [   5   ,   10  ]<- 0
PSI1  [   5   ,   11  ]<- 0
PSI1  [   5   ,   12  ]<- 0
PSI1  [   5   ,   13  ]<- 0

PSI1  [   6   ,   1   ]<- 0
PSI1  [   6   ,   2   ]<- 0
PSI1  [   6   ,   3   ]<- 0
PSI1  [   6   ,   4   ]<- 2*s[2]*(1-s[2]) # litter of 2, 1 cub survives
PSI1  [   6   ,   5   ]<- s[2]^2 # litter of 2, both cubs survive
```

```
PSI1  [   6   ,   6   ]<- 0
PSI1  [   6   ,   7   ]<- 0
PSI1  [   6   ,   8   ]<- (1- s[2]^2 -2*s[2]*(1-s[2])) #litter of 2, both cubs die

PSI1  [   6   ,   9   ]<- 0
PSI1  [   6   ,   10  ]<- 0
PSI1  [   6   ,   11  ]<- 0
PSI1  [   6   ,   12  ]<- 0
PSI1  [   6   ,   13  ]<- 0

PSI1  [   7   ,   1   ]<- 0
PSI1  [   7   ,   2   ]<- 0
PSI1  [   7   ,   3   ]<- 0
PSI1  [   7   ,   4   ]<- 0
PSI1  [   7   ,   5   ]<- 0
PSI1  [   7   ,   6   ]<- s[3] # litter of 1, yearling survives
PSI1  [   7   ,   7   ]<- 0
PSI1  [   7   ,   8   ]<- 0
PSI1  [   7   ,   9   ]<- (1-s[3] ) # litter of 1, yearling dies
PSI1  [   7   ,   10  ]<- 0
PSI1  [   7   ,   11  ]<- 0
PSI1  [   7   ,   12  ]<- 0
PSI1  [   7   ,   13  ]<- 0

PSI1  [   8   ,   1   ]<- 0
PSI1  [   8   ,   2   ]<- 0
PSI1  [   8   ,   3   ]<- 0
PSI1  [   8   ,   4   ]<- 0
PSI1  [   8   ,   5   ]<- 0
PSI1  [   8   ,   6   ]<- 2*s[4]*(1-s[4]) # litter of 2, 1 yearling survives
PSI1  [   8   ,   7   ]<- s[4]^2 # litter of 2, both yearlings survive
PSI1  [   8   ,   8   ]<- 0
PSI1  [   8   ,   9   ]<- (1- s[4]^2 -2*s[4]*(1-s[4])) #litter of 2, both yearlings die
PSI1  [   8   ,   10  ]<- 0
PSI1  [   8   ,   11  ]<- 0
PSI1  [   8   ,   12  ]<- 0
PSI1  [   8   ,   13  ]<- 0

PSI1  [   9   ,   1   ]<- 0
PSI1  [   9   ,   2   ]<- 0
PSI1  [   9   ,   3   ]<- 0
PSI1  [   9   ,   4   ]<- 0
PSI1  [   9   ,   5   ]<- 0
PSI1  [   9   ,   6   ]<- 0
PSI1  [   9   ,   7   ]<- 0
PSI1  [   9   ,   8   ]<- 0
PSI1  [   9   ,   9   ]<- 0
PSI1  [   9   ,   10  ]<- 1
PSI1  [   9   ,   11  ]<- 0
PSI1  [   9   ,   12  ]<- 0
PSI1  [   9   ,   13  ]<- 0

PSI1  [   10  ,   1   ]<- 0
```

```
PSI1  [   10  ,   2   ]<- 0
PSI1  [   10  ,   3   ]<- 0
PSI1  [   10  ,   4   ]<- 0
PSI1  [   10  ,   5   ]<- 0
PSI1  [   10  ,   6   ]<- 0
PSI1  [   10  ,   7   ]<- 0
PSI1  [   10  ,   8   ]<- 0
PSI1  [   10  ,   9   ]<- 0
PSI1  [   10  ,   10  ]<- 0
PSI1  [   10  ,   11  ]<- 1
PSI1  [   10  ,   12  ]<- 0
PSI1  [   10  ,   13  ]<- 0

PSI1  [   11  ,   1   ]<- 0
PSI1  [   11  ,   2   ]<- 0
PSI1  [   11  ,   3   ]<- 0
PSI1  [   11  ,   4   ]<- 0
PSI1  [   11  ,   5   ]<- 0
PSI1  [   11  ,   6   ]<- 0
PSI1  [   11  ,   7   ]<- 0
PSI1  [   11  ,   8   ]<- 0
PSI1  [   11  ,   9   ]<- 0
PSI1  [   11  ,   10  ]<- 0
PSI1  [   11  ,   11  ]<- 0
PSI1  [   11  ,   12  ]<- 1
PSI1  [   11  ,   13  ]<- 0

PSI1  [   12  ,   1   ]<- 0
PSI1  [   12  ,   2   ]<- 0
PSI1  [   12  ,   3   ]<- 0
PSI1  [   12  ,   4   ]<- 0
PSI1  [   12  ,   5   ]<- 0
PSI1  [   12  ,   6   ]<- 0
PSI1  [   12  ,   7   ]<- 0
PSI1  [   12  ,   8   ]<- 0
PSI1  [   12  ,   9   ]<- 0
PSI1  [   12  ,   10  ]<- 0
PSI1  [   12  ,   11  ]<- 0
PSI1  [   12  ,   12  ]<- 0
PSI1  [   12  ,   13  ]<- 1

# PSI2: breeding probabilities:
PSI2  [   1   ,   1   ]<- 1
PSI2  [   1   ,   2   ]<- 0
PSI2  [   1   ,   3   ]<- 0
PSI2  [   1   ,   4   ]<- 0
PSI2  [   1   ,   5   ]<- 0
PSI2  [   1   ,   6   ]<- 0
PSI2  [   1   ,   7   ]<- 0
PSI2  [   1   ,   8   ]<- 0
PSI2  [   1   ,   9   ]<- 0
PSI2  [   1   ,   10  ]<- 0
PSI2  [   1   ,   11  ]<- 0
```

```
PSI2  [   1   ,   12  ]<- 0
PSI2  [   1   ,   13  ]<- 0
PSI2  [   1   ,   14  ]<- 0
PSI2  [   1   ,   15  ]<- 0
PSI2  [   1   ,   16  ]<- 0

PSI2  [   2   ,   1   ]<- 0
PSI2  [   2   ,   2   ]<- 1
PSI2  [   2   ,   3   ]<- 0
PSI2  [   2   ,   4   ]<- 0
PSI2  [   2   ,   5   ]<- 0
PSI2  [   2   ,   6   ]<- 0
PSI2  [   2   ,   7   ]<- 0
PSI2  [   2   ,   8   ]<- 0
PSI2  [   2   ,   9   ]<- 0
PSI2  [   2   ,   10  ]<- 0
PSI2  [   2   ,   11  ]<- 0
PSI2  [   2   ,   12  ]<- 0
PSI2  [   2   ,   13  ]<- 0
PSI2  [   2   ,   14  ]<- 0
PSI2  [   2   ,   15  ]<- 0
PSI2  [   2   ,   16  ]<- 0

PSI2  [   3   ,   1   ]<- 0
PSI2  [   3   ,   2   ]<- 0
PSI2  [   3   ,   3   ]<- 1
PSI2  [   3   ,   4   ]<- 0
PSI2  [   3   ,   5   ]<- 0
PSI2  [   3   ,   6   ]<- 0
PSI2  [   3   ,   7   ]<- 0
PSI2  [   3   ,   8   ]<- 0
PSI2  [   3   ,   9   ]<- 0
PSI2  [   3   ,   10  ]<- 0
PSI2  [   3   ,   11  ]<- 0
PSI2  [   3   ,   12  ]<- 0
PSI2  [   3   ,   13  ]<- 0
PSI2  [   3   ,   14  ]<- 0
PSI2  [   3   ,   15  ]<- 0
PSI2  [   3   ,   16  ]<- 0

PSI2  [   4   ,   1   ]<- 0
PSI2  [   4   ,   2   ]<- 0
PSI2  [   4   ,   3   ]<- 0
PSI2  [   4   ,   4   ]<- 1
PSI2  [   4   ,   5   ]<- 0
PSI2  [   4   ,   6   ]<- 0
PSI2  [   4   ,   7   ]<- 0
PSI2  [   4   ,   8   ]<- 0
PSI2  [   4   ,   9   ]<- 0
PSI2  [   4   ,   10  ]<- 0
PSI2  [   4   ,   11  ]<- 0
PSI2  [   4   ,   12  ]<- 0
PSI2  [   4   ,   13  ]<- 0
```

```
PSI2  [   4   ,   14  ]<- 0
PSI2  [   4   ,   15  ]<- 0
PSI2  [   4   ,   16  ]<- 0

PSI2  [   5   ,   1   ]<- 0
PSI2  [   5   ,   2   ]<- 0
PSI2  [   5   ,   3   ]<- 0
PSI2  [   5   ,   4   ]<- 0
PSI2  [   5   ,   5   ]<- 1
PSI2  [   5   ,   6   ]<- 0
PSI2  [   5   ,   7   ]<- 0
PSI2  [   5   ,   8   ]<- 0
PSI2  [   5   ,   9   ]<- 0
PSI2  [   5   ,   10  ]<- 0
PSI2  [   5   ,   11  ]<- 0
PSI2  [   5   ,   12  ]<- 0
PSI2  [   5   ,   13  ]<- 0
PSI2  [   5   ,   14  ]<- 0
PSI2  [   5   ,   15  ]<- 0
PSI2  [   5   ,   16  ]<- 0

PSI2  [   6   ,   1   ]<- 0
PSI2  [   6   ,   2   ]<- 0
PSI2  [   6   ,   3   ]<- 0
PSI2  [   6   ,   4   ]<- 0
PSI2  [   6   ,   5   ]<- 0
PSI2  [   6   ,   6   ]<- 1
PSI2  [   6   ,   7   ]<- 0
PSI2  [   6   ,   8   ]<- 0
PSI2  [   6   ,   9   ]<- 0
PSI2  [   6   ,   10  ]<- 0
PSI2  [   6   ,   11  ]<- 0
PSI2  [   6   ,   12  ]<- 0
PSI2  [   6   ,   13  ]<- 0
PSI2  [   6   ,   14  ]<- 0
PSI2  [   6   ,   15  ]<- 0
PSI2  [   6   ,   16  ]<- 0

PSI2  [   7   ,   1   ]<- 0
PSI2  [   7   ,   2   ]<- 0
PSI2  [   7   ,   3   ]<- 0
PSI2  [   7   ,   4   ]<- 0
PSI2  [   7   ,   5   ]<- 0
PSI2  [   7   ,   6   ]<- 0
PSI2  [   7   ,   7   ]<- 1
PSI2  [   7   ,   8   ]<- 0
PSI2  [   7   ,   9   ]<- 0
PSI2  [   7   ,   10  ]<- 0
PSI2  [   7   ,   11  ]<- 0
PSI2  [   7   ,   12  ]<- 0
PSI2  [   7   ,   13  ]<- 0
PSI2  [   7   ,   14  ]<- 0
PSI2  [   7   ,   15  ]<- 0
```

```
PSI2  [   7   ,   16  ]<- 0

PSI2  [   8   ,   1   ]<- 0
PSI2  [   8   ,   2   ]<- 0
PSI2  [   8   ,   3   ]<- 0
PSI2  [   8   ,   4   ]<- 0
PSI2  [   8   ,   5   ]<- 0
PSI2  [   8   ,   6   ]<- 0
PSI2  [   8   ,   7   ]<- 0
PSI2  [   8   ,   8   ]<- beta[1]
PSI2  [   8   ,   9   ]<- 1-beta[1]
PSI2  [   8   ,   10  ]<- 0
PSI2  [   8   ,   11  ]<- 0
PSI2  [   8   ,   12  ]<- 0
PSI2  [   8   ,   13  ]<- 0
PSI2  [   8   ,   14  ]<- 0
PSI2  [   8   ,   15  ]<- 0
PSI2  [   8   ,   16  ]<- 0

PSI2  [   9   ,   1   ]<- 0
PSI2  [   9   ,   2   ]<- 0
PSI2  [   9   ,   3   ]<- 0
PSI2  [   9   ,   4   ]<- 0
PSI2  [   9   ,   5   ]<- 0
PSI2  [   9   ,   6   ]<- 0
PSI2  [   9   ,   7   ]<- 0
PSI2  [   9   ,   8   ]<- 0
PSI2  [   9   ,   9   ]<- 0
PSI2  [   9   ,   10  ]<- beta[2]
PSI2  [   9   ,   11  ]<- 1-beta[2]
PSI2  [   9   ,   12  ]<- 0
PSI2  [   9   ,   13  ]<- 0
PSI2  [   9   ,   14  ]<- 0
PSI2  [   9   ,   15  ]<- 0
PSI2  [   9   ,   16  ]<- 0

PSI2  [   10  ,   1   ]<- 0
PSI2  [   10  ,   2   ]<- 0
PSI2  [   10  ,   3   ]<- 0
PSI2  [   10  ,   4   ]<- 0
PSI2  [   10  ,   5   ]<- 0
PSI2  [   10  ,   6   ]<- 0
PSI2  [   10  ,   7   ]<- 0
PSI2  [   10  ,   8   ]<- 0
PSI2  [   10  ,   9   ]<- 0
PSI2  [   10  ,   10  ]<- 0
PSI2  [   10  ,   11  ]<- 0
PSI2  [   10  ,   12  ]<- beta[3]
PSI2  [   10  ,   13  ]<- 1-beta[3]
PSI2  [   10  ,   14  ]<- 0
PSI2  [   10  ,   15  ]<- 0
PSI2  [   10  ,   16  ]<- 0
```

```
PSI2  [   11  ,   1   ]<- 0
PSI2  [   11  ,   2   ]<- 0
PSI2  [   11  ,   3   ]<- 0
PSI2  [   11  ,   4   ]<- 0
PSI2  [   11  ,   5   ]<- 0
PSI2  [   11  ,   6   ]<- 0
PSI2  [   11  ,   7   ]<- 0
PSI2  [   11  ,   8   ]<- 0
PSI2  [   11  ,   9   ]<- 0
PSI2  [   11  ,   10  ]<- 0
PSI2  [   11  ,   11  ]<- 0
PSI2  [   11  ,   12  ]<- beta[3]
PSI2  [   11  ,   13  ]<- 1-beta[3]
PSI2  [   11  ,   14  ]<- 0
PSI2  [   11  ,   15  ]<- 0
PSI2  [   11  ,   16  ]<- 0

PSI2  [   12  ,   1   ]<- 0
PSI2  [   12  ,   2   ]<- 0
PSI2  [   12  ,   3   ]<- 0
PSI2  [   12  ,   4   ]<- 0
PSI2  [   12  ,   5   ]<- 0
PSI2  [   12  ,   6   ]<- 0
PSI2  [   12  ,   7   ]<- 0
PSI2  [   12  ,   8   ]<- 0
PSI2  [   12  ,   9   ]<- 0
PSI2  [   12  ,   10  ]<- 0
PSI2  [   12  ,   11  ]<- 0
PSI2  [   12  ,   12  ]<- 0
PSI2  [   12  ,   13  ]<- 0
PSI2  [   12  ,   14  ]<- beta[3]
PSI2  [   12  ,   15  ]<- 1-beta[3]
PSI2  [   12  ,   16  ]<- 0

PSI2  [   13  ,   1   ]<- 0
PSI2  [   13  ,   2   ]<- 0
PSI2  [   13  ,   3   ]<- 0
PSI2  [   13  ,   4   ]<- 0
PSI2  [   13  ,   5   ]<- 0
PSI2  [   13  ,   6   ]<- 0
PSI2  [   13  ,   7   ]<- 0
PSI2  [   13  ,   8   ]<- 0
PSI2  [   13  ,   9   ]<- 0
PSI2  [   13  ,   10  ]<- 0
PSI2  [   13  ,   11  ]<- 0
PSI2  [   13  ,   12  ]<- 0
PSI2  [   13  ,   13  ]<- 0
PSI2  [   13  ,   14  ]<- 0
PSI2  [   13  ,   15  ]<- 0
PSI2  [   13  ,   16  ]<- 1

# PSI3:litter size probabilities
PSI3  [   1   ,   1   ]<- 0
```

```
PSI3  [   1   ,   2   ]<- 1
PSI3  [   1   ,   3   ]<- 0
PSI3  [   1   ,   4   ]<- 0
PSI3  [   1   ,   5   ]<- 0
PSI3  [   1   ,   6   ]<- 0
PSI3  [   1   ,   7   ]<- 0
PSI3  [   1   ,   8   ]<- 0
PSI3  [   1   ,   9   ]<- 0
PSI3  [   1   ,   10  ]<- 0
PSI3  [   1   ,   11  ]<- 0
PSI3  [   1   ,   12  ]<- 0

PSI3  [   2   ,   1   ]<- 0
PSI3  [   2   ,   2   ]<- 0
PSI3  [   2   ,   3   ]<- 1
PSI3  [   2   ,   4   ]<- 0
PSI3  [   2   ,   5   ]<- 0
PSI3  [   2   ,   6   ]<- 0
PSI3  [   2   ,   7   ]<- 0
PSI3  [   2   ,   8   ]<- 0
PSI3  [   2   ,   9   ]<- 0
PSI3  [   2   ,   10  ]<- 0
PSI3  [   2   ,   11  ]<- 0
PSI3  [   2   ,   12  ]<- 0

PSI3  [   3   ,   1   ]<- 0
PSI3  [   3   ,   2   ]<- 0
PSI3  [   3   ,   3   ]<- 0
PSI3  [   3   ,   4   ]<- 1
PSI3  [   3   ,   5   ]<- 0
PSI3  [   3   ,   6   ]<- 0
PSI3  [   3   ,   7   ]<- 0
PSI3  [   3   ,   8   ]<- 0
PSI3  [   3   ,   9   ]<- 0
PSI3  [   3   ,   10  ]<- 0
PSI3  [   3   ,   11  ]<- 0
PSI3  [   3   ,   12  ]<- 0

PSI3  [   4   ,   1   ]<- 0
PSI3  [   4   ,   2   ]<- 0
PSI3  [   4   ,   3   ]<- 0
PSI3  [   4   ,   4   ]<- 0
PSI3  [   4   ,   5   ]<- 0
PSI3  [   4   ,   6   ]<- 0
PSI3  [   4   ,   7   ]<- 1
PSI3  [   4   ,   8   ]<- 0
PSI3  [   4   ,   9   ]<- 0
PSI3  [   4   ,   10  ]<- 0
PSI3  [   4   ,   11  ]<- 0
PSI3  [   4   ,   12  ]<- 0

PSI3  [   5   ,   1   ]<- 0
PSI3  [   5   ,   2   ]<- 0
```

```
PSI3  [   5   ,   3   ]<- 0
PSI3  [   5   ,   4   ]<- 0
PSI3  [   5   ,   5   ]<- 0
PSI3  [   5   ,   6   ]<- 0
PSI3  [   5   ,   7   ]<- 0
PSI3  [   5   ,   8   ]<- 1
PSI3  [   5   ,   9   ]<- 0
PSI3  [   5   ,   10  ]<- 0
PSI3  [   5   ,   11  ]<- 0
PSI3  [   5   ,   12  ]<- 0

PSI3  [   6   ,   1   ]<- 0
PSI3  [   6   ,   2   ]<- 0
PSI3  [   6   ,   3   ]<- 0
PSI3  [   6   ,   4   ]<- 0
PSI3  [   6   ,   5   ]<- 0
PSI3  [   6   ,   6   ]<- 0
PSI3  [   6   ,   7   ]<- 0
PSI3  [   6   ,   8   ]<- 0
PSI3  [   6   ,   9   ]<- 1
PSI3  [   6   ,   10  ]<- 0
PSI3  [   6   ,   11  ]<- 0
PSI3  [   6   ,   12  ]<- 0

PSI3  [   7   ,   1   ]<- 0
PSI3  [   7   ,   2   ]<- 0
PSI3  [   7   ,   3   ]<- 0
PSI3  [   7   ,   4   ]<- 0
PSI3  [   7   ,   5   ]<- 0
PSI3  [   7   ,   6   ]<- 0
PSI3  [   7   ,   7   ]<- 0
PSI3  [   7   ,   8   ]<- 0
PSI3  [   7   ,   9   ]<- 0
PSI3  [   7   ,   10  ]<- 1
PSI3  [   7   ,   11  ]<- 0
PSI3  [   7   ,   12  ]<- 0

PSI3  [   8   ,   1   ]<- 0
PSI3  [   8   ,   2   ]<- 0
PSI3  [   8   ,   3   ]<- 0
PSI3  [   8   ,   4   ]<- 0
PSI3  [   8   ,   5   ]<- gamma[1]
PSI3  [   8   ,   6   ]<- 1-gamma[1]
PSI3  [   8   ,   7   ]<- 0
PSI3  [   8   ,   8   ]<- 0
PSI3  [   8   ,   9   ]<- 0
PSI3  [   8   ,   10  ]<- 0
PSI3  [   8   ,   11  ]<- 0
PSI3  [   8   ,   12  ]<- 0

PSI3  [   9   ,   1   ]<- 0
PSI3  [   9   ,   2   ]<- 0
PSI3  [   9   ,   3   ]<- 0
```

```
PSI3  [   9   ,   4   ]<- 0
PSI3  [   9   ,   5   ]<- 0
PSI3  [   9   ,   6   ]<- 0
PSI3  [   9   ,   7   ]<- 0
PSI3  [   9   ,   8   ]<- 0
PSI3  [   9   ,   9   ]<- 0
PSI3  [   9   ,   10  ]<- 0
PSI3  [   9   ,   11  ]<- 1
PSI3  [   9   ,   12  ]<- 0

PSI3  [   10  ,   1   ]<- 0
PSI3  [   10  ,   2   ]<- 0
PSI3  [   10  ,   3   ]<- 0
PSI3  [   10  ,   4   ]<- 0
PSI3  [   10  ,   5   ]<- gamma[1]
PSI3  [   10  ,   6   ]<-  1-gamma[1]
PSI3  [   10  ,   7   ]<- 0
PSI3  [   10  ,   8   ]<- 0
PSI3  [   10  ,   9   ]<- 0
PSI3  [   10  ,   10  ]<- 0
PSI3  [   10  ,   11  ]<- 0
PSI3  [   10  ,   12  ]<- 0

PSI3  [   11  ,   1   ]<- 0
PSI3  [   11  ,   2   ]<- 0
PSI3  [   11  ,   3   ]<- 0
PSI3  [   11  ,   4   ]<- 0
PSI3  [   11  ,   5   ]<- 0
PSI3  [   11  ,   6   ]<- 0
PSI3  [   11  ,   7   ]<- 0
PSI3  [   11  ,   8   ]<- 0
PSI3  [   11  ,   9   ]<- 0
PSI3  [   11  ,   10  ]<- 0
PSI3  [   11  ,   11  ]<- 1
PSI3  [   11  ,   12  ]<- 0

PSI3  [   12  ,   1   ]<- 0
PSI3  [   12  ,   2   ]<- 0
PSI3  [   12  ,   3   ]<- 0
PSI3  [   12  ,   4   ]<- 0
PSI3  [   12  ,   5   ]<- gamma[2]
PSI3  [   12  ,   6   ]<- 1-gamma[2]
PSI3  [   12  ,   7   ]<- 0
PSI3  [   12  ,   8   ]<- 0
PSI3  [   12  ,   9   ]<- 0
PSI3  [   12  ,   10  ]<- 0
PSI3  [   12  ,   11  ]<- 0
PSI3  [   12  ,   12  ]<- 0

PSI3  [   13  ,   1   ]<- 0
PSI3  [   13  ,   2   ]<- 0
PSI3  [   13  ,   3   ]<- 0
PSI3  [   13  ,   4   ]<- 0
```

```
PSI3  [   13  ,   5   ]<- 0
PSI3  [   13  ,   6   ]<- 0
PSI3  [   13  ,   7   ]<- 0
PSI3  [   13  ,   8   ]<- 0
PSI3  [   13  ,   9   ]<- 0
PSI3  [   13  ,   10  ]<- 0
PSI3  [   13  ,   11  ]<- 1
PSI3  [   13  ,   12  ]<- 0

PSI3  [   14  ,   1   ]<- 0
PSI3  [   14  ,   2   ]<- 0
PSI3  [   14  ,   3   ]<- 0
PSI3  [   14  ,   4   ]<- 0
PSI3  [   14  ,   5   ]<- gamma[2]
PSI3  [   14  ,   6   ]<- 1-gamma[2]
PSI3  [   14  ,   7   ]<- 0
PSI3  [   14  ,   8   ]<- 0
PSI3  [   14  ,   9   ]<- 0
PSI3  [   14  ,   10  ]<- 0
PSI3  [   14  ,   11  ]<- 0
PSI3  [   14  ,   12  ]<- 0

PSI3  [   15  ,   1   ]<- 0
PSI3  [   15  ,   2   ]<- 0
PSI3  [   15  ,   3   ]<- 0
PSI3  [   15  ,   4   ]<- 0
PSI3  [   15  ,   5   ]<- 0
PSI3  [   15  ,   6   ]<- 0
PSI3  [   15  ,   7   ]<- 0
PSI3  [   15  ,   8   ]<- 0
PSI3  [   15  ,   9   ]<- 0
PSI3  [   15  ,   10  ]<- 0
PSI3  [   15  ,   11  ]<- 1
PSI3  [   15  ,   12  ]<- 0

PSI3  [   16  ,   1   ]<- 0
PSI3  [   16  ,   2   ]<- 0
PSI3  [   16  ,   3   ]<- 0
PSI3  [   16  ,   4   ]<- 0
PSI3  [   16  ,   5   ]<- 0
PSI3  [   16  ,   6   ]<- 0
PSI3  [   16  ,   7   ]<- 0
PSI3  [   16  ,   8   ]<- 0
PSI3  [   16  ,   9   ]<- 0
PSI3  [   16  ,   10  ]<- 0
PSI3  [   16  ,   11  ]<- 0
PSI3  [   16  ,   12  ]<- 1

# Matrix product for state-to-state transitions S
S[1:12,1:12] <- PHI[1:12,1:12] %*% PSI1[1:12,1:13] %*% PSI2[1:13,1:16]  %*% PSI3[1:16,1:12]


## Observation process: Define probabilities of E(t) given S(t).
```

```
#for initial capture, conditional on first capture
E0    [   1   ,   1   ]<- 0
E0    [   1   ,   2   ]<- 1
E0    [   1   ,   3   ]<- 0
E0    [   1   ,   4   ]<- 0
E0    [   1   ,   5   ]<- 0
E0    [   1   ,   6   ]<- 0
E0    [   1   ,   7   ]<- 0
E0    [   1   ,   8   ]<- 0
E0    [   1   ,   9   ]<- 0
E0    [   1   ,   10  ]<- 0
E0    [   1   ,   11  ]<- 0
E0    [   1   ,   12  ]<- 0

E0    [   2   ,   1   ]<- 0
E0    [   2   ,   2   ]<- 0
E0    [   2   ,   3   ]<- 1
E0    [   2   ,   4   ]<- 0
E0    [   2   ,   5   ]<- 0
E0    [   2   ,   6   ]<- 0
E0    [   2   ,   7   ]<- 0
E0    [   2   ,   8   ]<- 0
E0    [   2   ,   9   ]<- 0
E0    [   2   ,   10  ]<- 0
E0    [   2   ,   11  ]<- 0
E0    [   2   ,   12  ]<- 0

E0    [   3   ,   1   ]<- 0
E0    [   3   ,   2   ]<- 0
E0    [   3   ,   3   ]<- 0
E0    [   3   ,   4   ]<- 1
E0    [   3   ,   5   ]<- 0
E0    [   3   ,   6   ]<- 0
E0    [   3   ,   7   ]<- 0
E0    [   3   ,   8   ]<- 0
E0    [   3   ,   9   ]<- 0
E0    [   3   ,   10  ]<- 0
E0    [   3   ,   11  ]<- 0
E0    [   3   ,   12  ]<- 0

E0    [   4   ,   1   ]<- 0
E0    [   4   ,   2   ]<- 0
E0    [   4   ,   3   ]<- 0
E0    [   4   ,   4   ]<- 0
E0    [   4   ,   5   ]<- 1
E0    [   4   ,   6   ]<- 0
E0    [   4   ,   7   ]<- 0
E0    [   4   ,   8   ]<- 0
E0    [   4   ,   9   ]<- 0
E0    [   4   ,   10  ]<- 0
E0    [   4   ,   11  ]<- 0
E0    [   4   ,   12  ]<- 0
```

```
E0    [   5   ,   1   ]<- 0
E0    [   5   ,   2   ]<- 0
E0    [   5   ,   3   ]<- 0
E0    [   5   ,   4   ]<- 0
E0    [   5   ,   5   ]<- 0
E0    [   5   ,   6   ]<- 1
E0    [   5   ,   7   ]<- 0
E0    [   5   ,   8   ]<- 0
E0    [   5   ,   9   ]<- 0
E0    [   5   ,   10  ]<- 0
E0    [   5   ,   11  ]<- 0
E0    [   5   ,   12  ]<- 0

E0    [   6   ,   1   ]<- 0
E0    [   6   ,   2   ]<- 0
E0    [   6   ,   3   ]<- 0
E0    [   6   ,   4   ]<- 0
E0    [   6   ,   5   ]<- 0
E0    [   6   ,   6   ]<- 0
E0    [   6   ,   7   ]<- 1
E0    [   6   ,   8   ]<- 0
E0    [   6   ,   9   ]<- 0
E0    [   6   ,   10  ]<- 0
E0    [   6   ,   11  ]<- 0
E0    [   6   ,   12  ]<- 0

E0    [   7   ,   1   ]<- 0
E0    [   7   ,   2   ]<- 0
E0    [   7   ,   3   ]<- 0
E0    [   7   ,   4   ]<- 0
E0    [   7   ,   5   ]<- 0
E0    [   7   ,   6   ]<- 0
E0    [   7   ,   7   ]<- 0
E0    [   7   ,   8   ]<- 1
E0    [   7   ,   9   ]<- 0
E0    [   7   ,   10  ]<- 0
E0    [   7   ,   11  ]<- 0
E0    [   7   ,   12  ]<- 0

E0    [   8   ,   1   ]<- 0
E0    [   8   ,   2   ]<- 0
E0    [   8   ,   3   ]<- 0
E0    [   8   ,   4   ]<- 0
E0    [   8   ,   5   ]<- 0
E0    [   8   ,   6   ]<- 0
E0    [   8   ,   7   ]<- 0
E0    [   8   ,   8   ]<- 0
E0    [   8   ,   9   ]<- 1
E0    [   8   ,   10  ]<- 0
E0    [   8   ,   11  ]<- 0
E0    [   8   ,   12  ]<- 0

E0    [   9   ,   1   ]<- 0
```

```
E0    [   9   ,   2   ]<- 0
E0    [   9   ,   3   ]<- 0
E0    [   9   ,   4   ]<- 0
E0    [   9   ,   5   ]<- 0
E0    [   9   ,   6   ]<- 0
E0    [   9   ,   7   ]<- 0
E0    [   9   ,   8   ]<- 0
E0    [   9   ,   9   ]<- 0
E0    [   9   ,   10  ]<- 1
E0    [   9   ,   11  ]<- 0
E0    [   9   ,   12  ]<- 0

E0    [   10  ,   1   ]<- 0
E0    [   10  ,   2   ]<- 0
E0    [   10  ,   3   ]<- 0
E0    [   10  ,   4   ]<- 0
E0    [   10  ,   5   ]<- 0
E0    [   10  ,   6   ]<- 0
E0    [   10  ,   7   ]<- 0
E0    [   10  ,   8   ]<- 0
E0    [   10  ,   9   ]<- 0
E0    [   10  ,   10  ]<- 0
E0    [   10  ,   11  ]<- 1
E0    [   10  ,   12  ]<- 0

E0    [   11  ,   1   ]<- 0
E0    [   11  ,   2   ]<- 0
E0    [   11  ,   3   ]<- 0
E0    [   11  ,   4   ]<- 0
E0    [   11  ,   5   ]<- 0
E0    [   11  ,   6   ]<- 0
E0    [   11  ,   7   ]<- 0
E0    [   11  ,   8   ]<- 0
E0    [   11  ,   9   ]<- 0
E0    [   11  ,   10  ]<- 0
E0    [   11  ,   11  ]<- 0
E0    [   11  ,   12  ]<- 1

E0    [   12  ,   1   ]<- 1
E0    [   12  ,   2   ]<- 0
E0    [   12  ,   3   ]<- 0
E0    [   12  ,   4   ]<- 0
E0    [   12  ,   5   ]<- 0
E0    [   12  ,   6   ]<- 0
E0    [   12  ,   7   ]<- 0
E0    [   12  ,   8   ]<- 0
E0    [   12  ,   9   ]<- 0
E0    [   12  ,   10  ]<- 0
E0    [   12  ,   11  ]<- 0
E0    [   12  ,   12  ]<- 0

# departure probability of a2 offspring
for(i in 1:N){
```

```
  for(t in 1:(Years-1)){
    E1    [   1   ,   1   ,i,t]<- 1
    E1    [   1   ,   2   ,i,t]<- 0
    E1    [   1   ,   3   ,i,t]<- 0
    E1    [   1   ,   4   ,i,t]<- 0
    E1    [   1   ,   5   ,i,t]<- 0
    E1    [   1   ,   6   ,i,t]<- 0
    E1    [   1   ,   7   ,i,t]<- 0
    E1    [   1   ,   8   ,i,t]<- 0
    E1    [   1   ,   9   ,i,t]<- 0
    E1    [   1   ,   10,i,t]<-   0
    E1    [   1   ,   11,i,t]<-   0
    E1    [   1   ,   12,i,t]<-   0

    E1    [   2   ,   1   ,i,t]<- 0
    E1    [   2   ,   2   ,i,t]<- 1
    E1    [   2   ,   3   ,i,t]<- 0
    E1    [   2   ,   4   ,i,t]<- 0
    E1    [   2   ,   5   ,i,t]<- 0
    E1    [   2   ,   6   ,i,t]<- 0
    E1    [   2   ,   7   ,i,t]<- 0
    E1    [   2   ,   8   ,i,t]<- 0
    E1    [   2   ,   9   ,i,t]<- 0
    E1    [   2   ,   10,i,t]<-   0
    E1    [   2   ,   11,i,t]<-   0
    E1    [   2   ,   12,i,t]<-   0

    E1    [   3   ,   1   ,i,t]<- 0
    E1    [   3   ,   2   ,i,t]<- 0
    E1    [   3   ,   3   ,i,t]<- 1
    E1    [   3   ,   4   ,i,t]<- 0
    E1    [   3   ,   5   ,i,t]<- 0
    E1    [   3   ,   6   ,i,t]<- 0
    E1    [   3   ,   7   ,i,t]<- 0
    E1    [   3   ,   8   ,i,t]<- 0
    E1    [   3   ,   9   ,i,t]<- 0
    E1    [   3   ,   10,i,t]<-   0
    E1    [   3   ,   11,i,t]<-   0
    E1    [   3   ,   12,i,t]<-   0

    E1    [   4   ,   1   ,i,t]<- 0
    E1    [   4   ,   2   ,i,t]<- 0
    E1    [   4   ,   3   ,i,t]<- 0
    E1    [   4   ,   4   ,i,t]<- 1
    E1    [   4   ,   5   ,i,t]<- 0
    E1    [   4   ,   6   ,i,t]<- 0
    E1    [   4   ,   7   ,i,t]<- 0
    E1    [   4   ,   8   ,i,t]<- 0
    E1    [   4   ,   9   ,i,t]<- 0
    E1    [   4   ,   10,i,t]<-   0
    E1    [   4   ,   11,i,t]<-   0
    E1    [   4   ,   12,i,t]<-   0
```

```
E1    [   5   ,   1,i,t]<-    0
E1    [   5   ,   2,i,t]<-    0
E1    [   5   ,   3,i,t]<-    0
E1    [   5   ,   4,i,t]<-    0
E1    [   5   ,   5,i,t]<-    1
E1    [   5   ,   6,i,t]<-    0
E1    [   5   ,   7,i,t]<-    0
E1    [   5   ,   8,i,t]<-    0
E1    [   5   ,   9,i,t]<-    0
E1    [   5   ,   10,i,t]<-   0
E1    [   5   ,   11,i,t]<-   0
E1    [   5   ,   12,i,t]<-   0

E1    [   6   ,   1   ,i,t]<- 0
E1    [   6   ,   2   ,i,t]<- 0
E1    [   6   ,   3   ,i,t]<- 0
E1    [   6   ,   4   ,i,t]<- 0
E1    [   6   ,   5   ,i,t]<- 0
E1    [   6   ,   6   ,i,t]<- 1
E1    [   6   ,   7   ,i,t]<- 0
E1    [   6   ,   8   ,i,t]<- 0
E1    [   6   ,   9   ,i,t]<- 0
E1    [   6   ,   10,i,t]<-   0
E1    [   6   ,   11,i,t]<-   0
E1    [   6   ,   12,i,t]<-   0

E1    [   7   ,   1   ,i,t]<- 0
E1    [   7   ,   2   ,i,t]<- 0
E1    [   7   ,   3   ,i,t]<- 0
E1    [   7   ,   4   ,i,t]<- 0
E1    [   7   ,   5   ,i,t]<- 0
E1    [   7   ,   6   ,i,t]<- 0
E1    [   7   ,   7   ,i,t]<- 1
E1    [   7   ,   8   ,i,t]<- 0
E1    [   7   ,   9   ,i,t]<- 0
E1    [   7   ,   10,i,t]<-   0
E1    [   7   ,   11,i,t]<-   0
E1    [   7   ,   12,i,t]<-   0

E1    [   8   ,   1   ,i,t]<- 0
E1    [   8   ,   2   ,i,t]<- 0
E1    [   8   ,   3   ,i,t]<- 0
E1    [   8   ,   4   ,i,t]<- 0
E1    [   8   ,   5   ,i,t]<- 0
E1    [   8   ,   6   ,i,t]<- 0
E1    [   8   ,   7   ,i,t]<- 0
E1    [   8   ,   8   ,i,t]<- 1
E1    [   8   ,   9   ,i,t]<- 0
E1    [   8   ,   10,i,t]<-   0
E1    [   8   ,   11,i,t]<-   0
E1    [   8   ,   12,i,t]<-   0

E1    [   9   ,   1   ,i,t]<- 0
```

```
E1    [   9   ,   2   ,i,t]<- 0
E1    [   9   ,   3   ,i,t]<- 0
E1    [   9   ,   4   ,i,t]<- 0
E1    [   9   ,   5   ,i,t]<- 0
E1    [   9   ,   6   ,i,t]<- 0
E1    [   9   ,   7   ,i,t]<- 0
E1    [   9   ,   8   ,i,t]<- 0
E1    [   9   ,   9   ,i,t]<- 1-alpha[i,t+1]
E1    [   9   ,   10,i,t]<-   0
E1    [   9   ,   11,i,t]<-   alpha[i,t+1]
E1    [   9   ,   12,i,t]<-   0

E1    [   10  ,   1,i,t]<-    0
E1    [   10  ,   2,i,t]<-    0
E1    [   10  ,   3,i,t]<-    0
E1    [   10  ,   4,i,t]<-    0
E1    [   10  ,   5,i,t]<-    0
E1    [   10  ,   6,i,t]<-    0
E1    [   10  ,   7,i,t]<-    0
E1    [   10  ,   8,i,t]<-    0
E1    [   10  ,   9,i,t]<-    2*(1-alpha[i,t+1])*alpha[i,t+1]
E1    [   10  ,   10,i,t]<-   1 - (2*(1-alpha[i,t+1])*alpha[i,t+1]) - (alpha[i,t+1])^2
E1    [   10  ,   11,i,t]<-   (alpha[i,t+1])^2
E1    [   10  ,   12,i,t]<-   0

E1    [   11  ,   1   ,i,t]<- 0
E1    [   11  ,   2   ,i,t]<- 0
E1    [   11  ,   3   ,i,t]<- 0
E1    [   11  ,   4   ,i,t]<- 0
E1    [   11  ,   5   ,i,t]<- 0
E1    [   11  ,   6   ,i,t]<- 0
E1    [   11  ,   7   ,i,t]<- 0
E1    [   11  ,   8   ,i,t]<- 0
E1    [   11  ,   9   ,i,t]<- 0
E1    [   11  ,   10,i,t]<-   0
E1    [   11  ,   11,i,t]<-   1
E1    [   11  ,   12,i,t]<-   0

E1    [   12  ,   1   ,i,t]<- 0
E1    [   12  ,   2   ,i,t]<- 0
E1    [   12  ,   3   ,i,t]<- 0
E1    [   12  ,   4   ,i,t]<- 0
E1    [   12  ,   5   ,i,t]<- 0
E1    [   12  ,   6   ,i,t]<- 0
E1    [   12  ,   7   ,i,t]<- 0
E1    [   12  ,   8   ,i,t]<- 0
E1    [   12  ,   9   ,i,t]<- 0
E1    [   12  ,   10,i,t]<-   0
E1    [   12  ,   11,i,t]<-   0
E1    [   12  ,   12,i,t]<-   1

# for recapture probability
E2    [   1   ,   1   ,i,t]<- 1 -p
```

```
    E2    [   1   ,   2   ,i,t]<- p
    E2    [   1   ,   3   ,i,t]<- 0
    E2    [   1   ,   4   ,i,t]<- 0
    E2    [   1   ,   5   ,i,t]<- 0
    E2    [   1   ,   6   ,i,t]<- 0
    E2    [   1   ,   7   ,i,t]<- 0
    E2    [   1   ,   8   ,i,t]<- 0
    E2    [   1   ,   9   ,i,t]<- 0
    E2    [   1   ,   10,i,t]<-   0
    E2    [   1   ,   11,i,t]<-   0
    E2    [   1   ,   12,i,t]<-   0

    E2    [   2   ,   1   ,i,t]<- 1 -p
    E2    [   2   ,   2   ,i,t]<- 0
    E2    [   2   ,   3   ,i,t]<- p
    E2    [   2   ,   4   ,i,t]<- 0
    E2    [   2   ,   5   ,i,t]<- 0
    E2    [   2   ,   6   ,i,t]<- 0
    E2    [   2   ,   7   ,i,t]<- 0
    E2    [   2   ,   8   ,i,t]<- 0
    E2    [   2   ,   9   ,i,t]<- 0
    E2    [   2   ,   10,i,t]<-   0
    E2    [   2   ,   11,i,t]<-   0
    E2    [   2   ,   12,i,t]<-   0

    E2    [   3   ,   1   ,i,t]<- 1 -p
    E2    [   3   ,   2   ,i,t]<- 0
    E2    [   3   ,   3   ,i,t]<- 0
    E2    [   3   ,   4   ,i,t]<- p
    E2    [   3   ,   5   ,i,t]<- 0
    E2    [   3   ,   6   ,i,t]<- 0
    E2    [   3   ,   7   ,i,t]<- 0
    E2    [   3   ,   8   ,i,t]<- 0
    E2    [   3   ,   9   ,i,t]<- 0
    E2    [   3   ,   10,i,t]<-   0
    E2    [   3   ,   11,i,t]<-   0
    E2    [   3   ,   12,i,t]<-   0

    E2    [   4   ,   1   ,i,t]<- 1 -p
    E2    [   4   ,   2   ,i,t]<- 0
    E2    [   4   ,   3   ,i,t]<- 0
    E2    [   4   ,   4   ,i,t]<- 0
    E2    [   4   ,   5   ,i,t]<- p
    E2    [   4   ,   6   ,i,t]<- 0
    E2    [   4   ,   7   ,i,t]<- 0
    E2    [   4   ,   8   ,i,t]<- 0
    E2    [   4   ,   9   ,i,t]<- 0
    E2    [   4   ,   10,i,t]<-   0
    E2    [   4   ,   11,i,t]<-   0
    E2    [   4   ,   12,i,t]<-   0

    E2    [   5   ,   1,i,t]<-    1 -p
    E2    [   5   ,   2,i,t]<-    0
```

```
    E2    [   5   ,   3,i,t]<-    0
    E2    [   5   ,   4,i,t]<-    0
    E2    [   5   ,   5,i,t]<-    0
    E2    [   5   ,   6,i,t]<-    p
    E2    [   5   ,   7,i,t]<-    0
    E2    [   5   ,   8,i,t]<-    0
    E2    [   5   ,   9,i,t]<-    0
    E2    [   5   ,   10,i,t]<-   0
    E2    [   5   ,   11,i,t]<-   0
    E2    [   5   ,   12,i,t]<-   0

    E2    [   6   ,   1   ,i,t]<- 1 -p
    E2    [   6   ,   2   ,i,t]<- 0
    E2    [   6   ,   3   ,i,t]<- 0
    E2    [   6   ,   4   ,i,t]<- 0
    E2    [   6   ,   5   ,i,t]<- 0
    E2    [   6   ,   6   ,i,t]<- 0
    E2    [   6   ,   7   ,i,t]<- p
    E2    [   6   ,   8   ,i,t]<- 0
    E2    [   6   ,   9   ,i,t]<- 0
    E2    [   6   ,   10,i,t]<-   0
    E2    [   6   ,   11,i,t]<-   0
    E2    [   6   ,   12,i,t]<-   0

    E2    [   7   ,   1   ,i,t]<- 1 -p
    E2    [   7   ,   2   ,i,t]<- 0
    E2    [   7   ,   3   ,i,t]<- 0
    E2    [   7   ,   4   ,i,t]<- 0
    E2    [   7   ,   5   ,i,t]<- 0
    E2    [   7   ,   6   ,i,t]<- 0
    E2    [   7   ,   7   ,i,t]<- 0
    E2    [   7   ,   8   ,i,t]<- p
    E2    [   7   ,   9   ,i,t]<- 0
    E2    [   7   ,   10,i,t]<-   0
    E2    [   7   ,   11,i,t]<-   0
    E2    [   7   ,   12,i,t]<-   0

    E2    [   8   ,   1   ,i,t]<- 1 -p
    E2    [   8   ,   2   ,i,t]<- 0
    E2    [   8   ,   3   ,i,t]<- 0
    E2    [   8   ,   4   ,i,t]<- 0
    E2    [   8   ,   5   ,i,t]<- 0
    E2    [   8   ,   6   ,i,t]<- 0
    E2    [   8   ,   7   ,i,t]<- 0
    E2    [   8   ,   8   ,i,t]<- 0
    E2    [   8   ,   9   ,i,t]<- p
    E2    [   8   ,   10,i,t]<-   0
    E2    [   8   ,   11,i,t]<-   0
    E2    [   8   ,   12,i,t]<-   0

    E2    [   9   ,   1   ,i,t]<- 1-p
    E2    [   9   ,   2   ,i,t]<- 0
    E2    [   9   ,   3   ,i,t]<- 0
```

```
    E2    [   9   ,   4   ,i,t]<- 0
    E2    [   9   ,   5   ,i,t]<- 0
    E2    [   9   ,   6   ,i,t]<- 0
    E2    [   9   ,   7   ,i,t]<- 0
    E2    [   9   ,   8   ,i,t]<- 0
    E2    [   9   ,   9   ,i,t]<- 0
    E2    [   9   ,   10,i,t]<-   p
    E2    [   9   ,   11,i,t]<-   0
    E2    [   9   ,   12,i,t]<-   0

    E2    [   10  ,   1,i,t]<-    1-p
    E2    [   10  ,   2,i,t]<-    0
    E2    [   10  ,   3,i,t]<-    0
    E2    [   10  ,   4,i,t]<-    0
    E2    [   10  ,   5,i,t]<-    0
    E2    [   10  ,   6,i,t]<-    0
    E2    [   10  ,   7,i,t]<-    0
    E2    [   10  ,   8,i,t]<-    0
    E2    [   10  ,   9,i,t]<-    0
    E2    [   10  ,   10,i,t]<-   0
    E2    [   10  ,   11,i,t]<-   p
    E2    [   10  ,   12,i,t]<-   0

    E2    [   11  ,   1   ,i,t]<- 1 - p
    E2    [   11  ,   2   ,i,t]<- 0
    E2    [   11  ,   3   ,i,t]<- 0
    E2    [   11  ,   4   ,i,t]<- 0
    E2    [   11  ,   5   ,i,t]<- 0
    E2    [   11  ,   6   ,i,t]<- 0
    E2    [   11  ,   7   ,i,t]<- 0
    E2    [   11  ,   8   ,i,t]<- 0
    E2    [   11  ,   9   ,i,t]<- 0
    E2    [   11  ,   10,i,t]<-   0
    E2    [   11  ,   11,i,t]<-   0
    E2    [   11  ,   12,i,t]<-   p

    E2    [   12  ,   1   ,i,t]<- 1
    E2    [   12  ,   2   ,i,t]<- 0
    E2    [   12  ,   3   ,i,t]<- 0
    E2    [   12  ,   4   ,i,t]<- 0
    E2    [   12  ,   5   ,i,t]<- 0
    E2    [   12  ,   6   ,i,t]<- 0
    E2    [   12  ,   7   ,i,t]<- 0
    E2    [   12  ,   8   ,i,t]<- 0
    E2    [   12  ,   9   ,i,t]<- 0
    E2    [   12  ,   10,i,t]<-   0
    E2    [   12  ,   11,i,t]<-   0
    E2    [   12  ,   12,i,t]<-   0

    # Matrix product for offspring independence and recapture
    E[1:12,1:12,i,t] <- E1[1:12,1:12,i,t] %*% E2[1:12,1:12,i,t]
  }
}
```

```
## LIKELIHOOD

for (i in 1:N)  # for each individual
{
  # The estimated probabilities of initial states S0 are the proportions in each state at first captu
  alive[i,First[i]] ~ dcat(S0[1:12])
  mydata[i,First[i]] ~ dcat(E0[alive[i,First[i]],1:12])

  for (j in (First[i]+1):Years)
  {

    ## STATE EQUATIONS ##
    # draw S(t) given S(t-1)
    alive[i,j] ~ dcat(S[alive[i,j-1],1:12])

    ## OBSERVATION EQUATIONS ##
    # draw events E(t) given states S(t)

    mydata[i,j] ~ dcat(E[alive[i,j],1:12,i,j-1])

  }
}


## PRIORS
# capture probability
p ~ dunif(0,1)

# juveniles, subadults and adult survival
#for(i in 1:2){phi[i] ~ dunif(0,1)}
phi[1] ~ dunif(0,1)

# initial states
for (i in 1:10){ log(prop[i]) <- theta[i]
theta[i] ~ dnorm(0,1)}

# offspring survival
# litter survival n=2 offspring
l02 <- 1 -(1- s[2]^2 -2*s[2]*(1-s[2]))
l12 <- 1- (1- s[4]^2 -2*s[4]*(1-s[4]))
# indiviual offspring survival
#for(i in 1:4){s[i]~ dunif(0,1)}

for(i in 1:2){s[i]~ dunif(0,1)}
  # Set constraints
  for(u in 1:2){  X[u] ~ dunif(0,1)} # with X ~ U[0,1] then  (a + ( b - a ) * X)
  #so that s[1] < s[3] <phi[1] for litter of 1
  s[3] <- s[1] + (phi[1] - s[1]) * X[1]
  # and s[2] < s[4] < phi[1] for litters of 2
  s[4] <- s[2] + (phi[1] - s[2]) * X[2]

# Breeding probability
kappa ~ dunif(0,1)
```

```
  for(i in 1:3){beta[i]~ dunif(0,1)}

  # Litter size probability
  for(i in 1:2){gamma[i]~ dunif(0,1)}

} # end model

 ")
```

This model differs from the model used for the simulations in just a few points. It assumes breeding probability and litter size probability does not vary between successful breeders (states AS1 and AS2) and female without dependent offspring (state A) by setting $beta_3 = beta_4$ (in the code beta[3]) and $gamma_3 = gamma_4$ (in the code gamma[2]) . It also assumes that litter size probability is the same among failed breeders (loss of cub versus yearling litter), by setting $gamma_1 = gamma_2$ (in the code gamma[1]).

Run the jags model and save the results:

```
# Call JAGS from R
out <- jags(data=mydatax,inits=inits, parameters.to.save=params,
            model.file = 'Multieventmodel_FitresidentBeardata.txt',n.chains=2,n.iter=20000,n.burnin=900

save(out,file='noage_lb_phip.RData')
```

Analyse the results

Load useful packages:

```
library(jagsUI)
library(MCMCvis)
```

Load model results and print a summary :

```
load(file='Fit_beardata.RData')
MCMCsummary(out,round=2)
```

```
##              mean   sd   2.5%    50%   97.5% Rhat n.eff
## phi          0.93 0.01   0.92   0.93    0.95 1.00  2027
## s[1]         0.54 0.10   0.34   0.54    0.72 1.00  4400
## s[2]         0.51 0.05   0.41   0.51    0.62 1.00  4400
## s[3]         0.67 0.11   0.46   0.68    0.87 1.00  1458
## s[4]         0.80 0.09   0.59   0.82    0.93 1.01   422
## l02          0.76 0.05   0.65   0.76    0.85 1.00  4400
## l12          0.95 0.04   0.83   0.97    0.99 1.01   331
## kappa        0.12 0.08   0.02   0.11    0.30 1.00  4400
## beta[1]      0.09 0.06   0.01   0.07    0.23 1.00  2978
## beta[2]      0.58 0.21   0.19   0.57    0.96 1.00  4400
## beta[3]      0.52 0.04   0.43   0.52    0.61 1.00  1763
## gamma[1]     0.35 0.17   0.07   0.34    0.71 1.00  2652
## gamma[2]     0.40 0.05   0.30   0.40    0.51 1.00  4400
## p            0.25 0.01   0.22   0.25    0.27 1.01   182
## prop[1]      0.56 0.16   0.30   0.54    0.92 1.00  4400
## prop[2]      0.46 0.14   0.24   0.44    0.79 1.00  4400
## prop[3]      0.66 0.18   0.37   0.63    1.08 1.00  4400
## prop[4]      0.42 0.14   0.21   0.40    0.74 1.00  4400
## prop[5]      0.80 0.21   0.45   0.77    1.28 1.00  4400
```

```
## prop[6]      0.76  0.20    0.43    0.74    1.22 1.00  4400
## prop[7]      0.46  0.15    0.24    0.44    0.80 1.00  4400
## prop[8]      0.20  0.08    0.08    0.19    0.40 1.00  4400
## prop[9]      0.20  0.08    0.08    0.19    0.40 1.00  4400
## prop[10]     0.09  0.05    0.02    0.08    0.22 1.00  4400
## deviance 1611.02 25.81 1563.30 1609.45 1664.85 1.02   133
```

Check mixing of the chains and posterior distributions:

```
MCMCtrace(out,params = c('phi','s','l02','l12','kappa','beta','gamma','p'),pdf=FALSE)
```

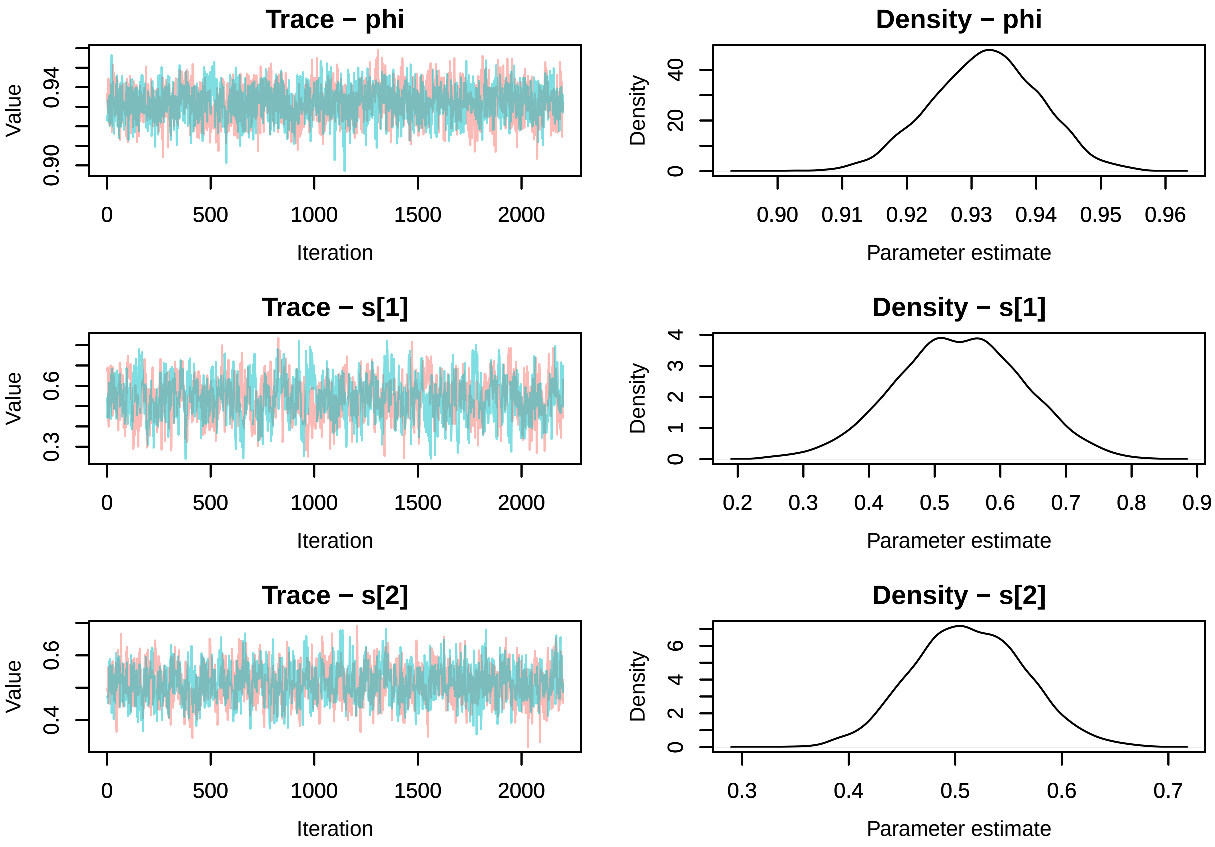

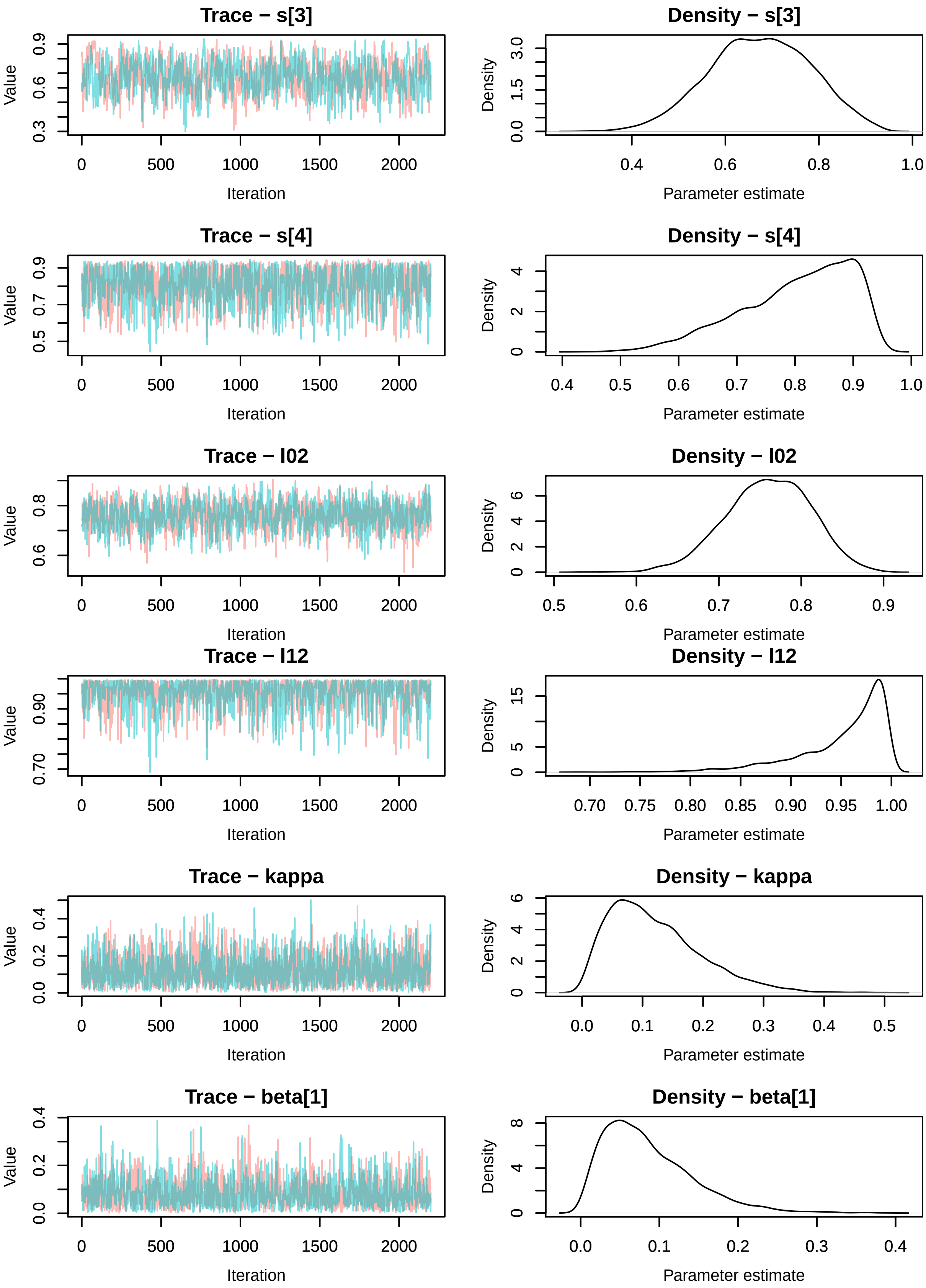
Trace − s[3]
Value
0.3
0.6
0.9
0
500
1000
1500
2000
Iteration
Density − s[3]
Density
0.0
1.5
3.0
0.4
0.6
0.8
1.0
Parameter estimate
Trace − s[4]
Value
0.5
0.7
0.9
0
500
1000
1500
2000
Iteration
Density − s[4]
Density
0
2
4
0.4
0.5
0.6
0.7
0.8
0.9
1.0
Parameter estimate
Trace − l02
Value
0.6
0.8
0
500
1000
1500
2000
Iteration
Density − l02
Density
0
2
4
6
0.5
0.6
0.7
0.8
0.9
Parameter estimate
Trace − l12
Value
0.70
0.90
0
500
1000
1500
2000
Iteration
Density − l12
Density
0
5
15
0.70
0.75
0.80
0.85
0.90
0.95
1.00
Parameter estimate
Trace − kappa
Value
0.0
0.2
0.4
0
500
1000
1500
2000
Iteration
Density − kappa
Density
0
2
4
6
0.0
0.1
0.2
0.3
0.4
0.5
Parameter estimate
Trace − beta[1]
Value
0.0
0.2
0.4
0
500
1000
1500
2000
Iteration
Density − beta[1]
Density
0
4
8
0.0
0.1
0.2
0.3
0.4
Parameter estimate

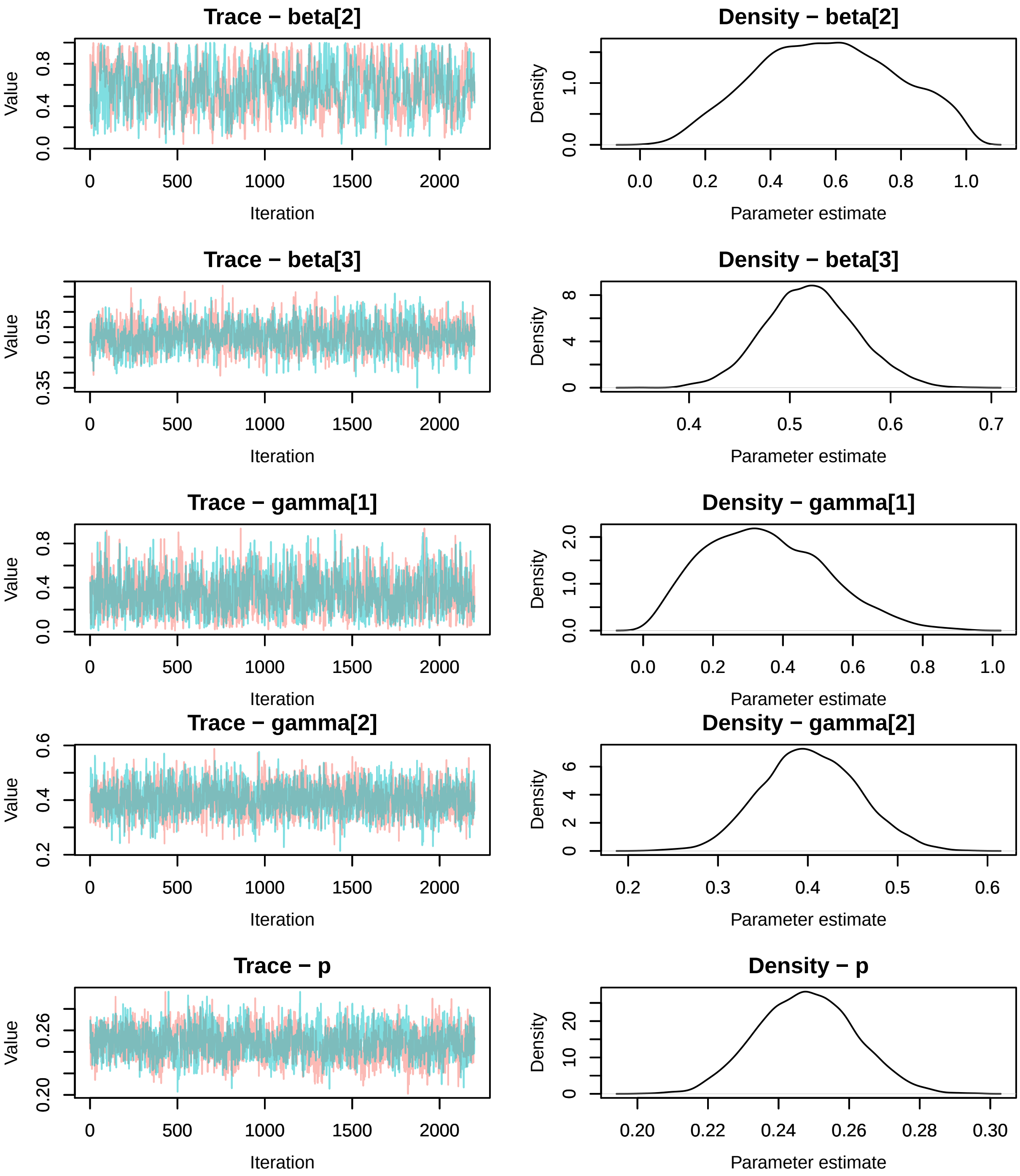

Plot a summary of the posterior distribution for each model parameter:

```
MCMCplot(out,
         params = c('phi','s','l02','l12','kappa','beta','gamma','p'),
         xlim = c(0, 1),
         xlab = 'Value',
         main = 'Estimates',
        labels = c(expression(phi),expression(s[1]),expression(s[2]),expression(s[3]),
                   expression(s[4]), expression(l[02]),expression(l[12]),expression(kappa),
                   expression(beta[1]),expression(beta[2]),expression(beta[3]),
```

```
                  expression(gamma[1]),expression(gamma[3]),expression(p)),
        col = 'black',
        sz_labels = 1.5,
        sz_med = 1.5,
        sz_thick = 4,
        sz_thin = 2,
        sz_ax = 4,
        sz_main_txt = 2)
```

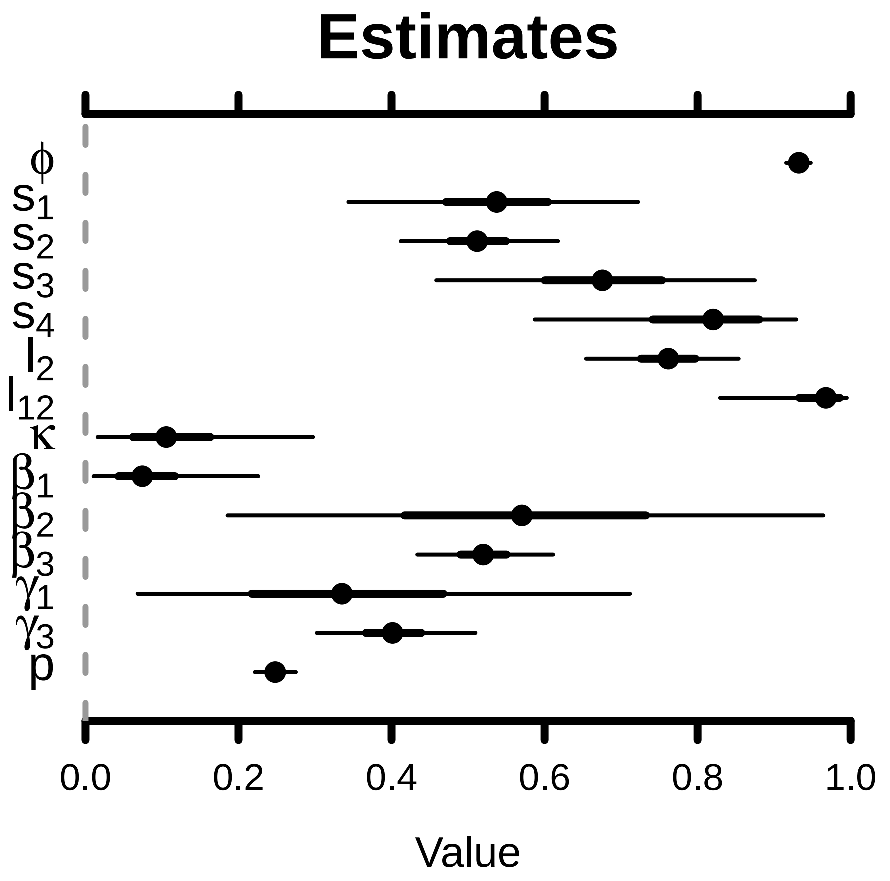

Dots represent posterior medians, thick lines represent 50 percent credible intervals while thin lines represent 95 percent credible intervals.

Calculate the probabilities of successfully raising 1 or 2 or 0 offspring to independence over a three-year period for an adult female without dependent offspring at the start of the period:

```
prx1 <- (out$sims.list$phi)^3 * out$sims.list$beta[,3] *
  (
    out$sims.list$gamma[,2] * out$sims.list$s[,1] * out$sims.list$s[,3]
    +
(1- out$sims.list$gamma[,2]) *
  (2 * out$sims.list$s[,2] * (1-out$sims.list$s[,2]) * out$sims.list$s[,3]
+ (out$sims.list$s[,2])^2 + 2 * out$sims.list$s[,4] * (1 -out$sims.list$s[,4])
)
)

prx2 <- (out$sims.list$phi)^3 * out$sims.list$beta[,3] *
  (1- out$sims.list$gamma[,2]) * (out$sims.list$s[,2])^2 * (out$sims.list$s[,4])^2
```

```r
prx0 <- 1 -prx1 -prx2
```

Make a table with the median value and 95% credible interval for the probabilities of successfully raising 1 or 2 or 0 offspring to independence over a three-year period for an adult female without dependent offspring at the start of the period:

```r
# Summary of results
tabres <- matrix(NA,3,3)
tabres[1,]<-quantile(prx0,probs=c(0.025,0.5,0.975))
tabres[2,]<-quantile(prx1,probs=c(0.025,0.5,0.975))
tabres[3,]<-quantile(prx2,probs=c(0.025,0.5,0.975))
rownames(tabres)=c("Pr(X=0)","Pr(X=1)","Pr(X=2)")
colnames(tabres)=c("Q 2.5%","Median","Q 97.5%")
round(tabres,2)
```

```
##         Q 2.5% Median Q 97.5%
## Pr(X=0)   0.57   0.67    0.76
## Pr(X=1)   0.20   0.29    0.38
## Pr(X=2)   0.02   0.04    0.07
```

References

Amstrup, S. C., & Durner, G. M. (1995). Survival rates of radio-collared female polar bears and their dependent young. Canadian Journal of Zoology, 73, 1312–1322. doi:10.1139/z95-155

Hornik, K., Leisch, F., & Zeileis, A. (2003). JAGS: A program for analysis of Bayesian graphical models using Gibbs sampling. In Proceedings of DSC (Vol. 2, No. 1).

Kellner, K. (2015). jagsUI: a wrapper around rjags to streamline JAGS analyses. R package version, 1(1).
